# CD38/ADP-ribose/TRPM2-mediated nuclear Ca^2+^ signaling is essential for hepatic gluconeogenesis in fasting and diabetes

**DOI:** 10.1038/s12276-023-01034-9

**Published:** 2023-07-03

**Authors:** So-Young Rah, Yeonsoo Joe, Jeongmin Park, Stefan W. Ryter, Chansu Park, Hun Taeg Chung, Uh-Hyun Kim

**Affiliations:** 1 Department of Biochemistry and National Creative Research Laboratory for Ca2+ Signaling Network, Jeonbuk National University, Medical School, Keum-am dong, Jeonju, 54907 Republic of Korea; 2 School of Biological Sciences, University of Ulsan, Ulsan, 44610 Republic of Korea; 3 Proterris Inc, Boston, MA 02118 USA; 4 Department of Biochemistry, School of Medicine, Wonkwang University, Iksan, 54538 Republic of Korea

**Keywords:** Type 2 diabetes, Calcium signalling

## Abstract

Hepatic glucose production by glucagon is crucial for glucose homeostasis during fasting, yet the underlying mechanisms remain incompletely delineated. Although CD38 has been detected in the nucleus, its function in this compartment is unknown. Here, we demonstrate that nuclear CD38 (nCD38) controls glucagon-induced gluconeogenesis in primary hepatocytes and liver in a manner distinct from CD38 occurring in the cytoplasm and lysosomal compartments. We found that the localization of CD38 in the nucleus is required for glucose production by glucagon and that nCD38 activation requires NAD^+^ supplied by PKCδ-phosphorylated connexin 43. In fasting and diabetes, nCD38 promotes sustained Ca^2+^ signals via transient receptor potential melastatin 2 (TRPM2) activation by ADP-ribose, which enhances the transcription of glucose-6 phosphatase and phosphoenolpyruvate carboxykinase 1. These findings shed light on the role of nCD38 in glucagon-induced gluconeogenesis and provide insight into nuclear Ca^2+^ signals that mediate the transcription of key genes in gluconeogenesis under physiological conditions.

## Diabetes: role of glucose-related protein explored

Improved understanding of how glucose levels are linked to a key protein in liver cells may lead to novel treatments for diabetes. During fasting, hepatocytes (liver cells) convert stored glycogen into glucose in response to the hormone glucagon hormone. Using mouse models, Uh-Hyun Kim (Jeonbuk National University, Jeonju), Hun Taeg Chung (University of Ulsan), in South Korea, and co-workers showed that the mammalian ADP-ribosyl cyclase protein CD38 localises in the nucleus of hepatocytes where it plays a previously unknown but essential role in glucose production. In fasting and diabetes, nuclear CD38 prompts the production of ADP-ribose, triggering changes essential for generating the sustained calcium ion signals vital for responding to glucagon. The team found that ADP-ribose production was increased in diabetic mice. Using a drug to decrease ADP-ribose effects reduced abnormally high glucose levels.

## Introduction

The liver is the primary organ in the maintenance of glucose homeostasis under fasting conditions^1^. During the early stages of fasting, glucagon secreted by pancreatic α-cells acts on hepatocytes to convert stored glycogen to glucose via glycogenolysis^2,3^. As fasting progresses, glucagon stimulates glucose production from noncarbohydrate precursors, such as amino acids or lactate, via gluconeogenesis, which then becomes the main contributor to hepatic glucose production^1,4^. In hepatic gluconeogenesis, glucose-6-phosphatase (G6Pase) and phosphoenolpyruvate carboxykinase 1 (Pck1) represent the key regulatory enzymes; their expression is closely regulated by Ca^2+^ signaling^5,6^. Glucagon increases cytosolic Ca^2+^ through the protein kinase-A (PKA)-mediated phosphorylation of the inositol 1,4,5-trisphosphate receptor (IP_3_R), leading to increased calcineurin activity and the subsequent dephosphorylation of the cAMP response element-binding protein (CREB) coactivator CRTC2^5^. We have previously demonstrated that glucagon induces CD38-mediated cyclic ADP-ribose (cADPR) production and sustained increases in cytosolic Ca^2+^, which in turn induce nuclear translocation of CRTC and gluconeogenic gene expression^6^.

CD38 is a mammalian ADP-ribosyl cyclase that catalyzes the synthesis of multiple Ca^2+^-mobilizing messengers: cADPR, nicotinic acid adenine dinucleotide phosphate (NAADP), and ADP-ribose (ADPR)^7^. Although CD38 was initially identified as a type II transmembrane ectoenzyme in the plasma membrane, it was later revealed that CD38 exists in various intracellular organelles, including endosomes, lysosomes, and the nucleus^8–10^. The nuclear localization of CD38 has been demonstrated in a variety of cell types^8,11,12^. CD38 is assimilated in the inner nuclear membrane with its catalytic site within the nucleoplasm, suggesting that its product, cADPR, activates ryanodine receptors (RyR) on the inner nuclear membrane to cause an increase in nucleoplasmic Ca^2+^^8^. However, the specific characterization of this nuclear form of CD38 (nCD38) remains controversial, and the mechanism of its activation also remains unknown.

The nucleus has an autonomous Ca^2+^ signaling system that can generate its own Ca^2+^ transients that modulate processes such as gene transcription^13^. However, much remains unknown regarding how such spatially-distinct Ca^2+^ signals achieve specificity for gene transcription and how these nuclear Ca^2+^ signals are regulated. Numerous studies have demonstrated that the nucleus possesses the biochemical machinery required to produce Ca^2+^-mobilizing messengers^13–15^. These Ca^2+^-mobilizing messengers, IP_3_ and cADPR, stimulate the release of Ca^2+^ from the nuclear envelope (NE) into the nucleoplasm *via* IP_3_R and RyR located on the inner membrane of the nucleus^16–18^. Moreover, Gerasimenko et al.^16^ described ATP-dependent accumulation of Ca^2+^ in the NE, indicating that the NE serves as a “storage facility” for Ca^2+^ influx into the nucleoplasm. Together, these findings indicate that nuclear Ca^2+^ signaling operates independently of cytosolic signaling, although how these processes interact is not yet fully understood.

In this study, we investigated the role of nCD38 in glucagon-mediated Ca^2+^ signaling as well as hepatic gluconeogenic gene expression. Our findings support the view that nCD38 in hepatocytes produces a novel Ca^2+^ signaling messenger, ADPR, in response to glucagon, and thereby plays a critical role in glucagon-induced gluconeogenic gene expression. We provide evidence that ADPR production is regulated by the transfer of the substrate NAD^+^ into the perinuclear space from the nucleoplasm via connexin43 (Cx43). Cx43 is in turn regulated by protein kinase C (PKC) δ-mediated phosphorylation. ADPR targets the transient receptor potential melastatin 2 (TRPM2) channel to gate Ca^2+^ from the NE into the nucleoplasm. Finally, we present evidence that ADPR production is upregulated in diabetic animals and that treating diabetic animals with an ADPR antagonist mitigates the abnormally increased glucose levels. Our findings emphasize that ADPR-mediated nuclear Ca^2+^ signaling is essential for hepatic gluconeogenesis under both normal physiological conditions and in diabetes.

## Materials and methods

### Animals

CD38 knockout mice (*Cd38*^−/−^; B6.129P2-Cd38tm/Lud) and B6. BKS(D)-Lepr^db^/J (*db*/*db*) mice were purchased from Jackson Laboratory (Bar Harbor, ME). TRPM2 KO mice were kindly provided by Yasuo Mori (Kyoto University, Japan). All experimental animals were used under a protocol approved by the institutional animal care and use committee at Jeonbuk National University Medical School (CBU 2014-00031).

### Reagents

Xestospongin C was obtained from Santa Cruz Biotechnology. Trans-Ned19, GF10923X, Go6976, rottlerin, and SKF96365 were obtained from Tocris Bioscience. 8-Br-ADPR and ara-2’-F-NAD were obtained from Biolog Life Science Institute. All other reagents were obtained from Sigma-Aldrich.

### Adenoviral constructs

Generation of NLS-R GECO Adenovirus: NLS R GECO adenovirus was constructed using NLS R GECO plasmid (Addgene, 32462)^19^. Generation of NLS-CD38 and NLS-Flag Cx43 Adenovirus: CD38 or Cx43 was amplified *via* PCR from pCMV-CD38 (Sino Biological Inc., MG50191-UT) or pCMV3- Flag Cx43 (Sino Biological Inc., G52427-NF) by using primers to add NLS, which was inserted into the pCMV/myc/nuc vector (Thermo Fisher Scientific, V82120). NLS-CD38 E230D and NLS-Flag Cx43 S368A were constructed by the addition of NLS *via* site-directed mutagenesis. The resulting construct was subcloned into the pENTR D-TOPO vector (Thermo Fisher Scientific, K240020), after which a recombinant vector was generated using the pAd/CMV/V5-DEST adenoviral expression vector system (Life Technologies, 43-0200). Recombinant adenovirus was amplified in HEK293A cells (Thermo Fisher Scientific, R70507) and purified by cesium chloride gradient ultracentrifugation.

### Primary hepatocyte culture and glucose production

Primary hepatocytes were isolated from 8- to 12-week-old male C57BL/6J mice or *db*/*db* mice as previously described^20^. Hepatocytes were transduced with adenoviral constructs 6 h after plating at a multiplicity of infection of 50 plaque-forming units per cell for 16 h, and experiments were performed 36 h after transduction. Glucose production assays were carried out as described^21^.

### In vivo imaging

Seven-week-old male C57BL/6 J mice were intravenously injected with 1 × 10^9^ p.f.u of CRE luciferase adenovirus (*db/db* male mice, 5 × 10^10^ p.f.u) from Vector Biolabs. After 3 days, 6 h-fasted mice were injected intraperitoneally with 100 μg/kg glucagon 1 h before imaging. Then, the mice were injected intraperitoneally with 150 mg/kg firefly d‐luciferin (LUCK-100, GoldBio, St Louis, MO, USA). After 10 min, mice were anesthetized and imaged at 15 min using the IVIS Luminar XR Imaging System (Caliper Life Sciences, Hopkinton, MA, USA).

### Pyruvate tolerance test and glycerol tolerance test

Mice were fasted for 19 h and injected intraperitoneally with sodium pyruvate or glycerol (2 g/kg body weight). Tail vein blood was sampled for glucose measurement at the indicated time points after injection. Blood glucose values were determined using a LifeScan automatic glucometer.

### Biochemical analysis

Plasma levels of insulin (ALPCO) and glucagon (MyBioSource), as well as glycogen content (BioAssay Systems), were assessed as per the manufacturer’s instructions.

### Isolation of nuclei

Intact nuclei from hepatocytes were isolated as previously described^17^. To prepare the outer membrane of the nucleus and nucleoplast (outer membrane removed nuclei), 2% (*w*/*v*) sodium citrate was added to the nuclear suspension, incubated for 1 h on ice while stirring and centrifuged for 15 min at 1000 g. The supernatant contained the outer membrane of the nucleus, and the pellet contained the nucleoplast.

### NAD glycohydrolase activity

NAD glycohydrolase activity was determined fluorometrically by using 1,N^6^-etheno-NAD as a substrate^22^.

### siRNA transfection

Hepatocytes were transfected with 100 nM siRNA specific for TRPM2 (Genolution), PKCδ, (Ambion, s71696), PLCδ1 (Ambion, s71805), PLCδ3 (Ambion, s91013), or control siRNA (Ambion, 4390843) using Lipofectamine RNAi MAX reagent according to the manufacturer’s instructions. Transfected hepatocytes were cultured for 36 h before experiments. The siRNA sequences were as follows: *TRPM2* siRNA-1, sense, 5’-GCACUCUGCAUACAAUCUAtt-3’, antisense, 5’-UAGAUUGUAUGCAGAGUGCgg-3’; *TRPM2* siRNA-2, sense, 5’-GCGUCUUCACUGAGUGCUAtt-3’, antisense, 5’-UAGCACUCAGUGAAGACGCgg-3’; *PKCδ* siRNA, sense, 5’-GAUUCAAGGUUUAUAACUAtt-3’, antisense, 5’-UAGUUAUAAACCUUGAAUCgg-3’; *PLCδ1* siRNA, sense, 5’-GCUACACUUUUACCUCUAAtt -3’, antisense, 5’-UUAGAGGUAAAAGUGUAGCca-3’; and *PLCδ3* siRNA, sense, 5’-GAGUGAGGAUGGUCGAAUUtt-3’, antisense, 5’-AAUUCGACCAUCCUCACUCcg-3’.

### Quantitative real-time PCR

Total cellular RNA was extracted from primary hepatocytes using the RNeasy kit (Qiagen, Valencia, CA). cDNA was synthesized by reverse transcription from 50 ng of total RNA using a cDNA synthesis kit (TaKaRa, RR037A). PCR was carried out on a 384-well plate using the ABI Prism 7900HT Sequence Detection System (Applied Biosystems) and SYBR Green Master Mix (Applied Biosystems, 4367659). Real-time PCR primers for mouse *G6pc*, *Pck1*, and *GAPDH* were as follows: *G6pc* (for, 5′-CGACTCGCTATCTCCAAGTGA-3′, and rev, 5′-GTTGAACCAGTCTCCGACCA-3′); *Pck1* (for, 5′-AAGCATTCAACGCCAGGTTC-3′, and rev, 5′-GGGCGAGTCTGTCAGTTCAAT-3′); *GAPDH* (for, 5′-CGTCCCGTAGACAAAATGGT-3′, and rev, 5′-TTGATGGCAACAATCTCCAC-3′). *GAPDH* mRNA expression was quantified to normalize all data. Mouse *β-Actin* and *PLC* isoforms primers were as follows: *β-Actin* (for, 5′- AAGGCCAACCGTGAAAAGATGACC-3′, and rev, 5′-ACCGCTCGTTGCCAATAGTGA TGA-3′); *PLCβ1* (for, 5′-AGACCTGGTGAACATTTCCCA-3′, and rev, 5′-ACAAGCCTCTAGTGCAGTTTC-3′); *PLCβ2* (for, 5′-CTCAACCCTGTTCTATTGCCC-3′, and rev, 5′-TCGGATACTCGTGACATCCAG-3′); *PLCβ3* (for, 5′-TGCCCAAGGACCCTAAGATTC-3′, and rev, 5′-GCTTCGTGTATGCTTTCCGC-3′); *PLCβ4* (for, 5′-AGTGCTAGAATGTTCCCTCATCA-3′, and rev, 5′- GAAGCCGATATTCACCAGATCC-3′); *PLCδ1* (for, 5′-CAAGGACCAGCGCAATACC-3′, and rev, 5′-CTTCCTGGCGTAGCTGTCATC-3′); *PLCδ3* (for, 5′- GGCTACGGGCACTGAAGAAG-3′, and rev, 5′-GCTGCACGAAGAATATGTGCTT-3′); *PLCδ4* (for, 5′-ATTCAAGACCTACTAGCCACTGA-3′, and rev, 5′- CTCCACCAGATAGCGCAACAA-3′); *PLCγ1* (for, 5′-TCTCGGGACTTTGACCGCTA-3′, and rev, 5′-CTCTCGGTTACGATCCACTGA-3′); *PLCγ2* (for, 5′-GTGGACACCCTTCCAGAATATG-3′, and rev, 5′-ACCTGCCGAGTCTCCATGAT-3′).

### Nuclear and cytoplasmic protein extraction

Nuclear and cytoplasmic proteins from hepatocytes or liver tissue were extracted using the NE-PER Nuclear and Cytoplasmic Extraction Kit (Thermo Fisher Scientific, 78833) according to the manufacturer’s instructions.

### Immunoblotting

Cells were lysed in RIPA buffer (50 mM Tris–HCl, pH 7.4, 150 mM NaCl, 1% sodium deoxycholate, 0.1% SDS, and 1% NP 40 supplemented with phosphatase and protease inhibitor cocktail, Roche). Lysates were boiled in Laemmli sample buffer for 10 min, separated by SDS-polyacrylamide gel electrophoresis, transferred to PVDF membranes, and probed with primary antibodies. We used antibodies for the following proteins: pCREB (Ser133) (Cell Signaling, 9198), pCaMKII (Cell Signaling, 3361), pCaMKIV (Santa Cruz, sc-28443-R), CREB (Cell Signaling, 9197), CaMKII (Cell Signaling, 3362), CaMKIV (Cell Signaling, 4032), PARP-1 (Santa Cruz, sc-53643), Actin (Merck Millipore, MAB1501), pCx43(Ser368) (Sigma-Aldrich, SAB4300504), Cx43 (Cell Signaling, 3512), Flag (Sigma-Aldrich, F7425), Lamin B1 (Cell Signaling, 12586), PKCδ (Cell Signaling, 9616), TRPM2 (Novus, NB110-81601), Na^+^K^+^-ATPase (Novus, NB300-146), Calregulin (Santa Cruz, sc-7431), Nesprin 3 (MyBioSource, MBS2535184), Myc (Invitrogen, 46-0603), CD38 (Santa Cruz, sc-7049), PLCβ1 (Santa Cruz, sc-5291), PLCβ3 (Santa Cruz, sc-133231), PLCβ4 (Santa Cruz, sc-404), PLCδ1 (Santa Cruz, sc-365811), PLCδ3 (Santa Cruz, sc-514912), PLCδ4 (Santa Cruz, sc-373875), PLCγ1 (Santa Cruz, sc-7290), PLCγ2 (Santa Cruz, sc-5283), HSP90 (Cell Signaling, 4874), GAPDH (Santa Cruz, sc-166574), and LAMP-1 (BD Biosciences, 553792). After incubation with secondary antibodies conjugated with horseradish peroxidase (Cell Signaling), chemiluminescence was detected by using the LAS4000 system (GE Healthcare). Western blot densitometric quantification was performed using ImageJ (NIH, Bethesda, MD).

### Immunofluorescence

Hepatocytes were grown on collagen-coated confocal dishes and fixed with 3.8% paraformaldehyde for 20 min, after which they were washed three times with ice-cold PBS. To permeabilize, cells and nuclei were treated with 0.25% Triton X-100 in PBS for 10 min. After blocking with 3% BSA, 0.25% Triton X-100, and PBS at RT for 1 h, samples were incubated overnight at 4 °C with the indicated antibodies: TRPM2 (Novus, NB110-81601), CD38 (Thermo Fisher Scientific, 14-0381-85), PKCδ (Santa Cruz, sc-8402), Cx43 (Santa Cruz, sc-13558), PLCδ1 (Santa Cruz, sc-365811), PLCδ3 (Santa Cruz, sc-514912), or Lamin B1 (Santa Cruz, sc-6216). Alexa Fluor-conjugated secondary antibodies (546 donkey anti-rabbit antibody, Thermo Fisher Scientific, A10040; 555 donkey anti-goat antibody, Thermo Fisher Scientific, A-21432; 488 donkey anti-rat antibody, Thermo Fisher Scientific, A-21208; 488 donkey anti-mouse antibody, Thermo Fisher Scientific, A-21202; or 488 donkey anti-rabbit antibody, Thermo Fisher Scientific, A-21206) were incubated at 1:500 dilutions in the presence of 1% BSA at RT for 1 h. The nuclei were stained with DAPI (Thermo Fisher Scientific, 62248). Cells and nuclei were visualized with a Zeiss LSM510 Axiovert 200 M laser-scanning confocal microscope.

### Measurement of [Ca^2+^]_i_

Hepatocytes infected with adenovirus regarding GCaMP6m (Vector Biolabs, 1909) and NLS-R-GECO for 24 h were subsequently serum-starved for 16 h in Medium 199, after which changes in [Ca^2+^]_i_ were determined at 488 nm excitation/530 nm emission and 543 nm excitation/560 nm emission using a confocal microscope (Nikon, Japan). Isolated nuclei were loaded as reported^23,24^. The membrane-impermeant Ca^2+^ dye Calcium Green Dextran (Thermo Fisher Scientific, C3713) was loaded into the nucleoplasm, while the membrane permeant Ca^2+^ dye Fluo-4 AM (Thermo Fisher Scientific, F14201) was loaded into the nuclear envelope. Ca^2+^ imaging in single isolated nuclei was performed using Nikon confocal microscopy.

### Measurement of NAD, [ADPR]_i,_ and [cADPR]_i_

NAD and ADPR levels were measured using LC-MS/MS as described previously^25^, and cADPR was measured by the cycling method described previously^26^.

### Statistical analysis

Statistical analyses were performed using GraphPad Prism 8. Data were analyzed by Student’s unpaired *t* test and one-way ANOVA followed by Tukey’s multiple comparison test. Data are represented as the mean ± standard error of the mean (SEM). The number of independent experiments and information about the statistical details and methods are shown in the relevant figure legends. A value of *P* < 0.05 was considered significant.

## Results

### Gluconeogenesis by glucagon is dependent on nuclear CD38

**Fig. 1:**
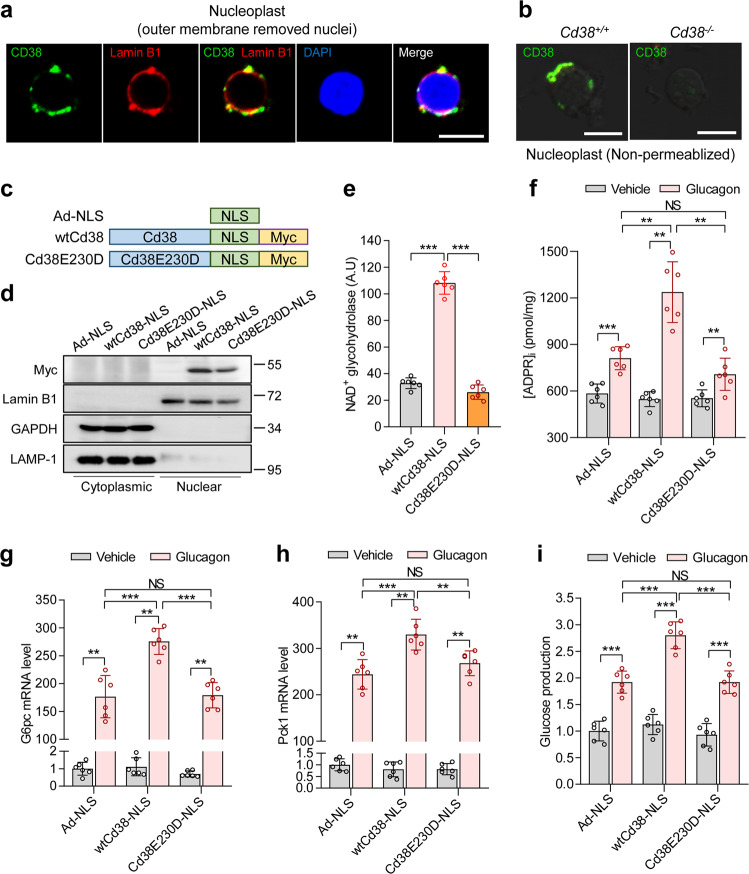
Nuclear CD38 is involved in gluconeogenesis by glucagon. **a** After isolating nuclei with sucrose ultracentrifugation, the outer membrane of the nuclei was separated by treatment with sodium citrate. Nucleoplasts were fixed with 3.8% paraformaldehyde for 20 min, permeabilized, and then stained with anti-mouse CD38 antibody (green). Lamin B1 was used as a marker for the inner membrane of the nucleus. The nucleus was stained with DAPI (blue). Scale bar, 5 μm. **b** After nuclei were isolated from *Cd38*^*+/+*^ or *Cd38*^−*/*−^ mouse primary hepatocytes, the outer membrane of the nucleus was removed by sodium citrate treatment. The nucleoplasts (outer membrane-deprived nuclei) were stained with an anti-mouse CD38 antibody (C90). Scale bar, 5 μm. **c** Adenoviral vector constructs of Control (Ad-NLS), catalytic active-nCD38 (wtCd38), and catalytic inactive-nCD38 (Cd38E230D). **d** Hepatocytes were transduced with adenoviral vectors expressing Ad-NLS, wtCd38-NLS, or Cd38E230D-NLS at an MOI of 50 for 24 h, and then cytoplasmic and nuclear extracts were isolated. Immunoblotting was performed to detect Myc, LAMP-1 (a marker of lysosomes), GAPDH (a cytoplasmic marker), and Lamin B1 (a nuclear marker). **e** NAD glycohydrolase activity. **f** ADPR levels in hepatocytes treated with glucagon (100 nM) for 30 sec. **g**, **h** qRT-PCR of *G6pc* (**g**) and *Pck1* (**h**) after incubation with glucagon (100 nM) for 4 h. **i** Glucose production after incubation with glucagon (100 nM) for 5 h. **e**–**i** *n* = 6 independent experiments, mean values ± SEMs are shown, and *P* values were calculated by one-way ANOVA followed by Tukey’s multiple comparison test. **P < 0.01, ***P < 0.001, and not significant (NS). For **a**, **b**, **d**, representative images of three independent experiments are shown.

CD38 is ubiquitously expressed in various cellular organelles, including the nucleus^8,27^. Although nCD38 has been found to regulate nuclear Ca^2+^ homeostasis^8,27^, the role of nCD38 in physiological functions remains unknown. First, we verified the existence of CD38 in the nucleus of primary hepatocytes. Confocal microscopy using anti-CD38 antisera revealed perinuclear immunofluorescence with ring-like labeling around the nucleus, which co-localized with Lamin B1 (Fig. 1a). Furthermore, CD38 was primarily located in the inner membrane of the nucleus but not the outer nuclear membrane (Supplementary Fig. 1a, b). In addition, nCD38 was detected in primary hepatocytes isolated from CD38 wild-type (*Cd38*^+/+^) but not CD38 knockout (*Cd38*^−/−^) mice (Fig. 1b). To further investigate the role of nCD38 in glucagon-induced hepatic gluconeogenesis, we overexpressed nCD38 in primary hepatocytes using adenovirus encoding a nuclear localizing signal (NLS)-fused CD38 (Ad-wtCd38-NLS). We also constructed an adenovirus encoding an NLS-fused catalytically inactive mutant CD38E230D (Ad-Cd38E230D-NLS) as a negative control (Fig. 1c). Western blot analyses revealed the expression of our constructs only in the nuclear fractions (Fig. 1d). CD38 overexpression from Ad-wtCd38-NLS resulted in robust NADase activity, whereas Ad-Cd38E230D-NLS-transfected cells did not exhibit any significant change in NADase activity compared to control Ad-NLS overexpressing cells (Fig. 1e). nCD38 overexpression led to further increases in ADPR production in response to glucagon relative to control Ad-NLS infected cells, which were not evident in cells overexpressing mutant nCD38 (Fig. 1f). However, nCD38 overexpression did not further increase cADPR production in response to glucagon (Supplementary Fig. 1d). In addition, to test the glucagon-induced ADPR increase in the nucleus, we measured the ADPR level in the nucleoplast, a nucleus stripped of its outer membrane, after treating nCD38-overexpressed primary hepatocytes with glucagon. ADPR production was increased in the nucleoplast of nCD38-overexpressed hepatocytes in response to glucagon when compared to the control Ad-NLS infected cells (Supplementary Fig. 1e). Next, to determine the function of nCD38 in hepatic glucose metabolism, the expression of the *G6pc* and *Pck1* genes encoding G6Pase and phosphoenolpyruvate carboxykinase 1, which are the major rate-limiting enzymes for the glucose-generating pathway^28^, was analyzed after glucagon treatment of hepatocytes. The increase in nCD38 by transfection with Ad-wtCd38-NLS enhanced the levels of *G6pc* and *Pck1* mRNA in the presence of glucagon relative to control Ad-NLS expressing cells, while Ad-Cd38E230D-NLS expression did not elicit this response (Fig. 1g, h). Consistently, the increased expression of nCD38, but not mutated nCD38, significantly promoted glucose production relative to control Ad-NLS expressing cells (Fig. 1i). Furthermore, we confirmed that the overexpression of nCD38 in *Cd38*^−/−^ hepatocytes via Ad-wtCd38-NLS significantly increased *G6pc* and *Pck1* mRNA levels in response to glucagon (Supplementary Fig. 1f, g). Therefore, these findings suggest that nCD38 may be involved in the regulation of gluconeogenesis-related gene expression and glucose metabolism.

### CD38 activation in the nucleus requires Cx43 phosphorylation by PKCδ

**Fig. 2:**
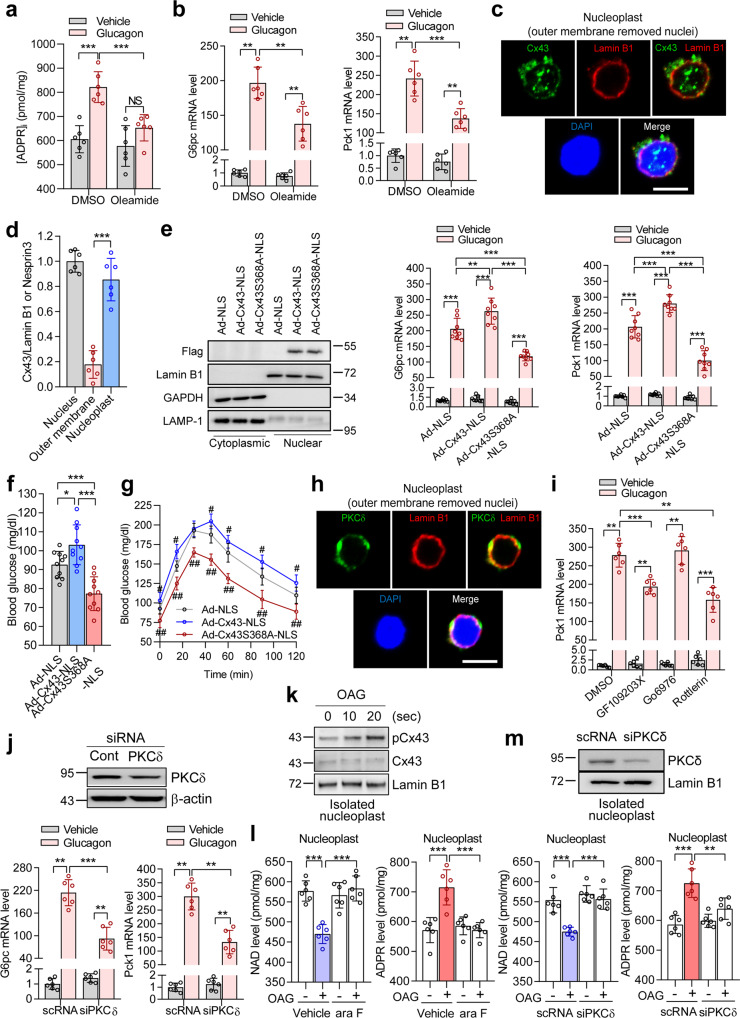
Cx43 regulates NAD^+^ transport to CD38 in the perinuclear space via phosphorylation by PKCδ. **a** Glucagon-induced ADPR production after preincubation with oleamide (50 μM) for 30 min. *n* = 6 independent experiments. ***P < 0.001 and not significant (NS). **b** Effect of oleamide on glucagon-induced mRNA expression of *G6pc* and *Pck1*. *n* = 6 indepe*n*dent experiments. **P < 0.01, ***P < 0.001. **c** After isolating nuclei, nucleoplasts were stained with anti-Cx43 antibody (green). Lamin B1 was used as a marker for the inner membrane of the nucleus. The nucleus was stained with DAPI (blue). Scale bar, 5 μm. **d** The bar graph represents the localization of Cx43. *n* = 6 independent experiments. ***P < 0.001. **e** Hepatocytes were transduced with adenoviral vectors expressing Ad-Cx43-NLS or Ad-Cx43^S368A^-NLS at an MOI of 50 for 24 h and then assayed for immunoblot analysis for Ad-NLS, Ad-Cx43-NLS, or Ad-Cx43^S368A^-NLS and qRT-PCR of *G6pc* and *Pck1* in hepatocytes infected with Cx43 WT or S368A before and after treatment with glucagon. *n* = 8 independent experiments. **P < 0.01, ***P < 0.001. **f**, **g** Blood glucose levels during fasting (**f**) and after pyruvate challenge (**g**) in mice after treatment with Ad-NLS, Ad-Cx43-NLS, or Ad-Cx43^S368A^-NLS. *n* = 10 mice per group. *P < 0.05, **P < 0.01, ***P < 0.001, ^#^*P* < 0.05; Ad-NLS *vs*. Ad-Cx43-NLS, ^##^*P* < 0.001; Ad-Cx43-NLS *vs*. Ad-Cx43^S368A^-NLS. **h** Nucleoplasts were stained with anti-PKCδ antibody (green). Lamin B1 was used as a marker for the inner membrane of the nucleus. The nucleus was stained with DAPI (blue). Scale bar, 5 μm. **i** Glucagon-induced *G6pc* and *Pck1* mRNA levels. GF109203X (1 μM), Go6976 (1 μM), or rottlerin (3 μM) was preincubated for 30 min. *n* = 6 independent experiments. **P < 0.01, ***P < 0.001. **j** Effects of PKCδ KD on glucagon-induced *G6pc* and *Pck1* gene expression. Immunoblotting of PKCδ and β-actin (upper). *n* = 6 independent experiments. **P < 0.01, ***P < 0.001. **k** Nucleoplasts were incubated with OAG for the indicated times. Immunoblotting for pCx43 (S368), Cx43, and Lamin B1. **l** NAD and ADPR levels after nucleoplasts were incubated with OAG (100 μM) for 30 sec. Ara-2’-F-NAD (200 nM) was preincubated for 30 min. *n* = 6 independent experiments. ***P < 0.001. **m** NAD and ADPR levels in PKCδ-knockdown nucleoplasts. OAG (100 μM) was incubated for 30 sec. Immunoblotting of PKCδ and Lamin B1 (upper). *n* = 6 independent experiments. **P < 0.01, ***P < 0.001. Data are represented as the mean ± SEM. Statistics were determined by one-way ANOVA followed by Tukey’s multiple comparison test except **d** where unpaired *t* test was used. For confocal images and immunoblotting, representative images of three independent experiments are shown.

Given that nCD38 is constitutively active and exists in a type II orientation on the inner membrane of the nucleus (Fig. 1b), we assumed that the regulation of nCD38 activity in the perinuclear space occurred through substrate availability. To determine whether Cx43, a known NAD^+^ transporter^29^, was involved in glucagon-induced nuclear Ca^2+^ signaling, we examined the effect of the Cx43 inhibitor oleamide on glucagon-induced ADPR production. Oleamide abolished glucagon-induced ADPR production as well as *G6pc* and *Pck1* mRNA expression levels (Fig. 2a, b).

Cx43 is regulated through Ser^368^ phosphorylation *via* PKC^30^. We explored whether Ser^368^ of Cx43 is phosphorylated during glucagon signaling in hepatocytes. Glucagon-induced Ser^368^ phosphorylation occurred as early as 1 min after stimulation (Supplementary Fig. 2a), which was also observed in isolated nuclei (Supplementary Fig. 2b). In addition, using confocal analysis, we confirmed the existence of Cx43 in the inner membrane of the nucleus (Fig. 2c, d and Supplementary Fig. 1c). To confirm the importance of Ser^368^ phosphorylation in glucagon signaling, we generated an adenovirus encoding an NLS-fused Cx43S368A mutant (Supplementary Fig. 2c). Western blot analyses revealed the expression of our constructs only in the nuclear fractions (Fig. 2e). Overexpression of nuclear-targeting Cx43 S368A in hepatocytes decreased glucagon-induced gluconeogenic gene expression levels compared to overexpression of wild-type nuclear-targeting Cx43 (Fig. 2e). Furthermore, the overexpression of nuclear-targeting Cx43S368A in mice *via* adenovirus resulted in decreased blood glucose levels in both the fasting state and in response to a pyruvate or glycerol challenge test when compared to levels from mice expressing wild-type Cx43 (Fig. 2f, g and Supplementary Fig. 2d). Western blot analyses of liver tissues revealed the expression of our constructs only in the nuclear fractions (Supplementary Fig. 2e). As described above, body weight, food intake, plasma insulin, and plasma glucagon showed minimal differences between the control and experimental groups (Supplementary Fig. 2f). In line with blood glucose data, hepatic *G6pc* and *Pck1* mRNA expression levels were lower in mice injected with the nuclear-targeting Cx43 S368A adenovirus (Supplementary Fig. 2g).

Because nuclear Cx43 inhibition resulted in a reduced response to glucagon in hepatic *G6pc* and *Pck1* mRNA expression, we compared liver glycogen content and found that the overexpression of nuclear-targeting Cx43S368A in mice resulted in significantly higher liver glycogen content, as opposed to mice expressing nuclear-targeting wild-type Cx43 (Supplementary Fig. 2h). These data further indicate that phosphorylation of Serine 368 in nuclear Cx43 affects plasma glucose levels, the conversion of pyruvate into glucose, and the gene expression of gluconeogenic enzymes.

As PKCδ is responsible for Ser^368^ phosphorylation^31^, we tested whether PKCδ was involved in glucagon-induced signaling. First, we confirmed that PKCδ is located in the inner membrane of the nucleus (Fig. 2h). Subsequently, we found that glucagon-induced *Pck1* mRNA expression was inhibited by both a broad-range PKC inhibitor, GF109203X, and the PKCδ inhibitor rottlerin but not by the PKCα/PKCβ inhibitor, Go6976 (Fig. 2i). PKCδ involvement in glucagon-induced *G6pc* and *Pck1* mRNA expression levels was confirmed by PKCδ knockdown (KD) experiments (Fig. 2j). Furthermore, we measured NAD^+^ and ADPR levels in the nucleoplast preparation before and after treatment with diacylglycerol (DAG) to activate Cx43. Treating the nucleoplast with 1-oleoyl-2-acetyl-sn-glycerol (OAG), a synthetic diacylglycerol analog that induces Cx43 phosphorylation, resulted in reduced NAD^+^ levels while increasing ADPR levels (Fig. 2k, l). Pretreatment with ara-F-NAD, a membrane-impermeable CD38 inhibitor, significantly inhibited OAG-induced ADPR production (Fig. 2l). PKCδ-KD nucleoplasts showed a significant decrease in OAG-induced ADPR production and an increase in NAD levels compared with control nucleoplasts (Fig. 2m). Based on these results, we concluded that Cx43 plays a role in regulating NAD^+^ transport to CD38, the catalytic site of which faces the perinuclear space.

### Nuclear CD38-mediated ADPR is required for the increase in nuclear Ca^2+^

**Fig. 3:**
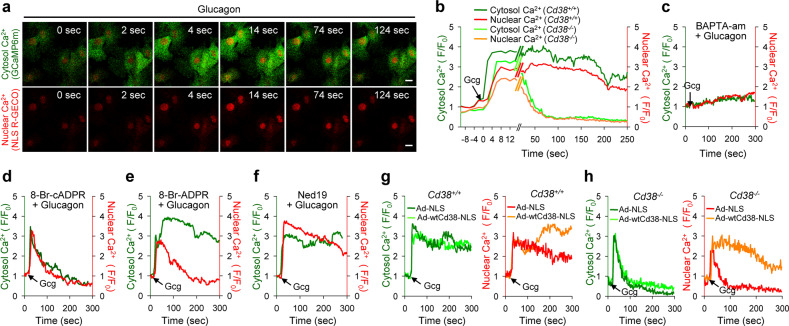
Nuclear CD38-mediated ADPR is essential for glucagon-induced nuclear Ca^2+^ signals. **a**, **b** Hepatocytes were infected with GCaMP6m and NLS-R-GECO adenoviruses, and Ca^2+^ signals were recorded. The time point where 100 nM glucagon (Gcg) is added is indicated by the arrow. Scale bar, 10 μm. **c** Cytosolic and nuclear Ca^2+^ responses to glucagon after preincubation with BAPTA-am (10 μM). **d**–**f** Cytosolic and nuclear Ca^2+^ responses to glucagon after incubation with various inhibitors of Ca^2+^ second messengers [8-Br-cADPR (100 μM, **d**), 8-Br-ADPR (100 μM, **e**), or Ned19 (10 μM, **f**)]. **g**, **h** Cytosolic and nuclear Ca^2+^ responses to glucagon after overexpression of nCD38 using wtCd38-NLS adenovirus in *Cd38*^*+/+*^ hepatocytes (**g**) or *Cd38*^−*/*−^ hepatocytes (**h**). *n* = 20 cells for each condition in **b**–**h**.

Previously we revealed that CD38 is involved in glucagon-induced cytosolic Ca^2+^ signaling in hepatocytes and produces a Ca^2+^ second messenger, cADPR^6^. In addition, oleamide inhibited glucagon-induced cADPR production (Supplementary Fig. 2i), which suggests its regulation by Cx43. As nCD38 can also produce Ca^2+^ second messengers through NAD^+^ glycohydrolase activity, we investigated whether nuclear Ca^2+^ signals are increased by nCD38 in glucagon-treated primary hepatocytes. To simultaneously measure the Ca^2+^ changes in hepatocyte cytosol and nucleus after glucagon treatment, we infected hepatocytes with adenovirus carrying the genetically-encoded fluorescent Ca^2+^ indicators, GCaMP6m and NLS-R-GECO. Increases in nuclear Ca^2+^ concentrations ([Ca^2+^]_nu_) lagged a few seconds behind glucagon treatment when compared to cytosolic Ca^2+^ levels, which increased immediately following exposure to glucagon, indicating that nuclear Ca^2+^ signals follow Ca^2+^ fluctuations in the cytosol (Fig. 3a, b). When cytosolic Ca^2+^ was chelated by pretreatment with BAPTA-AM, the glucagon-induced increase in [Ca^2+^]_nu_ was completely ablated (Fig. 3c), reinforcing the idea that nuclear Ca^2+^ signals are dependent on their cytosolic counterparts. As CD38-mediated cytosolic Ca^2+^ signals are involved in glucagon-induced gluconeogenesis in hepatocytes^6^, we also examined the possibility that nuclear Ca^2+^ signals may be dependent on CD38. Unlike primary hepatocytes of *Cd38*^+/+^ mice, glucagon-induced sustained Ca^2+^ signals in the nucleus as well as in the cytosol were absent in hepatocytes from *Cd38*^−/−^ mice (Fig. 3b). Pretreating Cd38^−/−^primary hepatocytes with xestospongin C (XeC; an inhibitor of IP_3_R) completely abolished glucagon-induced Ca^2+^ signals, including the initial signals, in the cytosol and the nucleus, but XeC only inhibited the sustained Ca^2+^ signal and not the initial Ca^2+^ signal in both the cytosol and nucleus in *Cd38*^+/+^ primary hepatocytes (Supplementary Fig. 3a). These findings indicate that glucagon-induced Ca^2+^ signals in both compartments are dependent on IP_3_ and Ca^2+^ second messenger(s) generated by CD38. We then sought to determine which Ca^2+^ second messengers are involved in glucagon-induced Ca^2+^ changes by using various antagonistic analogs. Glucagon-induced sustained Ca^2+^ signals in the cytosol and the nucleus were blocked by treatment with either XeC or 8-Br-cADPR (an antagonistic analog of cADPR) (Supplementary Fig. 3a, d), suggesting that IP_3_ and cADPR act as the first messengers in the initial Ca^2+^ signaling in both the cytosol and the nucleus of hepatocytes in response to glucagon treatment. These findings also indicate that both IP_3_- and cADPR-mediated Ca^2+^ signals are prerequisites for these glucagon-induced sustained Ca^2+^ signals. Intriguingly, 8-Br-ADPR (an antagonistic ADPR analog) only inhibited sustained Ca^2+^ signals in the nucleus (Fig. 3e). Ned19 (an NAADP antagonist) had no effect on glucagon-induced Ca^2+^ signaling in either the cytosol or the nucleus (Fig. 3f). These findings indicate that initial Ca^2+^ signals in the cytosol and the nucleus are dependent on IP_3_ and cADPR and that ADPR plays a role in the later phase of Ca^2+^ signaling in the nucleus.

Given that sustained Ca^2+^ signals in the cytosol and the nucleus were abolished by an IP_3_R blocker and cADPR antagonist (Supplementary Fig. 3a, d), we inferred that sustained Ca^2+^ signals require extracellular Ca^2+^ influx, which may be mediated through store-operated Ca^2+^ entry (SOCE) following Ca^2+^ store depletion by both IP_3_ and cADPR. As expected, glucagon-induced, sustained nuclear, and cytosolic Ca^2+^ signals were completely abolished under Ca^2+^-free conditions and by the SOCE inhibitor SKF 96365 (Supplementary Fig. 3b, c). These results suggest that both IP_3_ and cADPR-mediated cytosolic Ca^2+^ signals induce SOCE, leading to ADPR-mediated nuclear sustained Ca^2+^ signals.

To further examine the roles of nCD38 in glucagon-induced Ca^2+^ signaling, we measured Ca^2+^ changes in nCD38-overexpressing *Cd38*^*+/+*^ hepatocytes or *Cd38*^−*/*−^ hepatocytes. Overexpression of nCD38 increased glucagon-mediated nuclear sustained Ca^2+^ signals but not cytosolic Ca^2+^ signals in *Cd38*^*+/+*^ hepatocytes or *Cd38*^−*/*−^ hepatocytes (Fig. 3g, h). These results demonstrate that nCD38-produced ADPR is essential for glucagon-induced nuclear sustained Ca^2+^ signals.

### Nuclear Ca^2+^ influx requires nuclear TRPM2 activated by ADPR

**Fig. 4:**
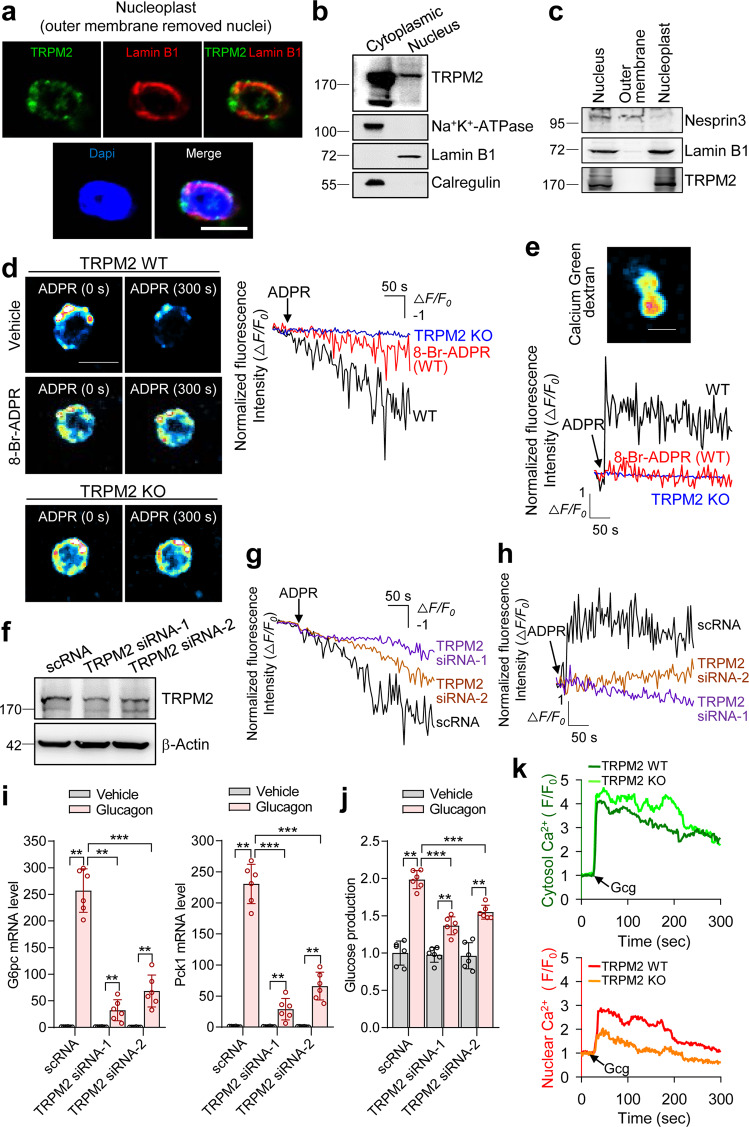
ADPR activates TRPM2 for nuclear Ca^2+^ influx from perinuclear Ca^2+^ stores. **a** Nucleoplasts were stained with anti-TRPM2 antibody (green). Lamin B1 was used as a marker for the inner membrane of the nucleus. The nuclei were stained with DAPI (blue). Scale bar, 5 μm. **b** Hepatocytes were separated into the cytoplasm and nucleus. TRPM2 localization was analyzed by immunoblot using antibodies against each marker: plasma membrane (Na^+^K^+^-ATPase), nucleus (Lamin B1), and endoplasmic reticulum (Calregulin). **c** Immunoblot for TRPM2, Nesprin3 (a marker for the outer membrane of the nucleus), and Lamin B1 (a marker for the inner membrane of the nucleus). **d**, **e** Changes in perinuclear Ca^2+^ levels were measured with Fluo-4 AM (**d**), and changes in the nucleoplasm Ca^2+^ levels were measured with Calcium Green dextran, a membrane-impermeant Ca^2+^ dye (**e**) after preincubation with 8-Br-ADPR (100 μM) for 30 min before treatment with ADPR (100 μM) in intact nuclei isolated from WT or TRPM2 KO hepatocytes. *n* = 5 nuclei for each condition. Scale bar, 5 μm. **f** Immunoblotting of TRPM2 expression after TRPM2 knockdown by siRNA. **g**, **h** Isolated nuclei after TRPM2 KD were measured for changes in perinuclear Ca^2+^ levels (**g**) and in nucleoplasm Ca^2+^ levels (**h**). *n* = 5 nuclei for each condition. **i**, **j** Effects of TRPM2 KD on glucagon-induced *G6pc* and *Pck1* gene expression (**i**) and glucose production (**j**). *n* = 6 independent experiments. **P < 0.01, ***P < 0.001. **k** Cytosolic and nuclear Ca^2+^ responses to glucagon in TRPM2 WT hepatocytes or TRPM2 KO hepatocytes. *n* = 20 cells for each condition in **k**. The time point where 100 nM glucagon (Gcg) was added is indicated by the *arrow*. Data are represented as the mean ± SEM. Statistics were determined by one-way ANOVA followed by Tukey’s multiple comparison test. For **a**–**c**, and **f** representative images of three independent experiments are shown.

ADPR activates TRPM2, a Ca^2+^-permeable nonselective cation channel, to elicit Ca^2+^ influx or Ca^2+^ release from intracellular stores^32–34^. We examined whether TRPM2 was located in the nucleus of primary hepatocytes. Confocal microscopy revealed perinuclear immunofluorescence with ring-like labeling around the nucleus (Fig. 4a). TRPM2 was also detected in both nuclear and cytoplasmic fractions (Fig. 4b). Specifically, the TRPM2 channel is likely localized to the inner membrane, but not the outer membrane, of the nucleus (Fig. 4c and Supplementary Fig. 1c). We further examined whether glucagon-induced nuclear Ca^2+^ signals were involved with the TRPM2 channel and perinuclear Ca^2+^ stores. To this end, we loaded hepatocyte nuclei with either the membrane-permeable Fluo-4 AM Ca^2+^ probe to measure Ca^2+^ in the perinuclear region or membrane-impermeable Calcium Green Dextran to measure Ca^2+^ in the nucleoplasm^18,23^. Intriguingly, confocal microscopy revealed that ADPR-induced reciprocal Ca^2+^ exchanges between the perinuclear area and the nucleoplasm, showing a decrease in perinuclear Ca^2+^ concentration and an increase in nucleoplasm Ca^2+^ concentration (Fig. 4d, e). The addition of ADPR led to decreased perinuclear Ca^2+^ levels, which correlated with an increase in nucleoplasm Ca^2+^. This is consistent with the previous finding that the perinuclear envelope serves as a Ca^2+^ store for the nucleoplasm^23^. 8-Br-ADPR completely blocked ADPR-induced Ca^2+^ exchange between the two spaces (Fig. 4d, e). Moreover, ADPR failed to induce Ca^2+^ efflux in hepatocytes from TRPM2 knockout (KO) mice (Fig. 4d, e). These findings suggest that ADPR activates the TRPM2 channel to induce Ca^2+^ efflux from perinuclear Ca^2+^ stores into the nucleoplasm to be utilized for nuclear Ca^2+^ signals in primary hepatocytes. These findings were corroborated by TRPM2 KD experiments using TRPM2-targeting siRNAs, showing that TRPM2 KD blocks ADPR-induced Ca^2+^ flux, as well as gluconeogenic gene transcription and glucose production (Fig. 4f–j). Furthermore, we measured both cytosol and nuclear Ca^2+^ changes in TRPM2 KO hepatocytes in response to glucagon. TRPM2 KO hepatocytes showed inhibition of nuclear Ca^2+^ signaling but not cytosolic Ca^2+^ signaling in response to glucagon compare to TRPM2 WT hepatocytes (Fig. 4k). These results indicate that TRPM2 is specifically involved in glucagon-induced nuclear Ca^2+^ signaling for hepatocyte gluconeogenesis, although TRPM2 is located in the lysosome.

### Nuclear Ca^2+^ increased by CD38/ADPR promotes gluconeogenesis via the CaMKII/CaMKIV/CREB pathway

**Fig. 5:**
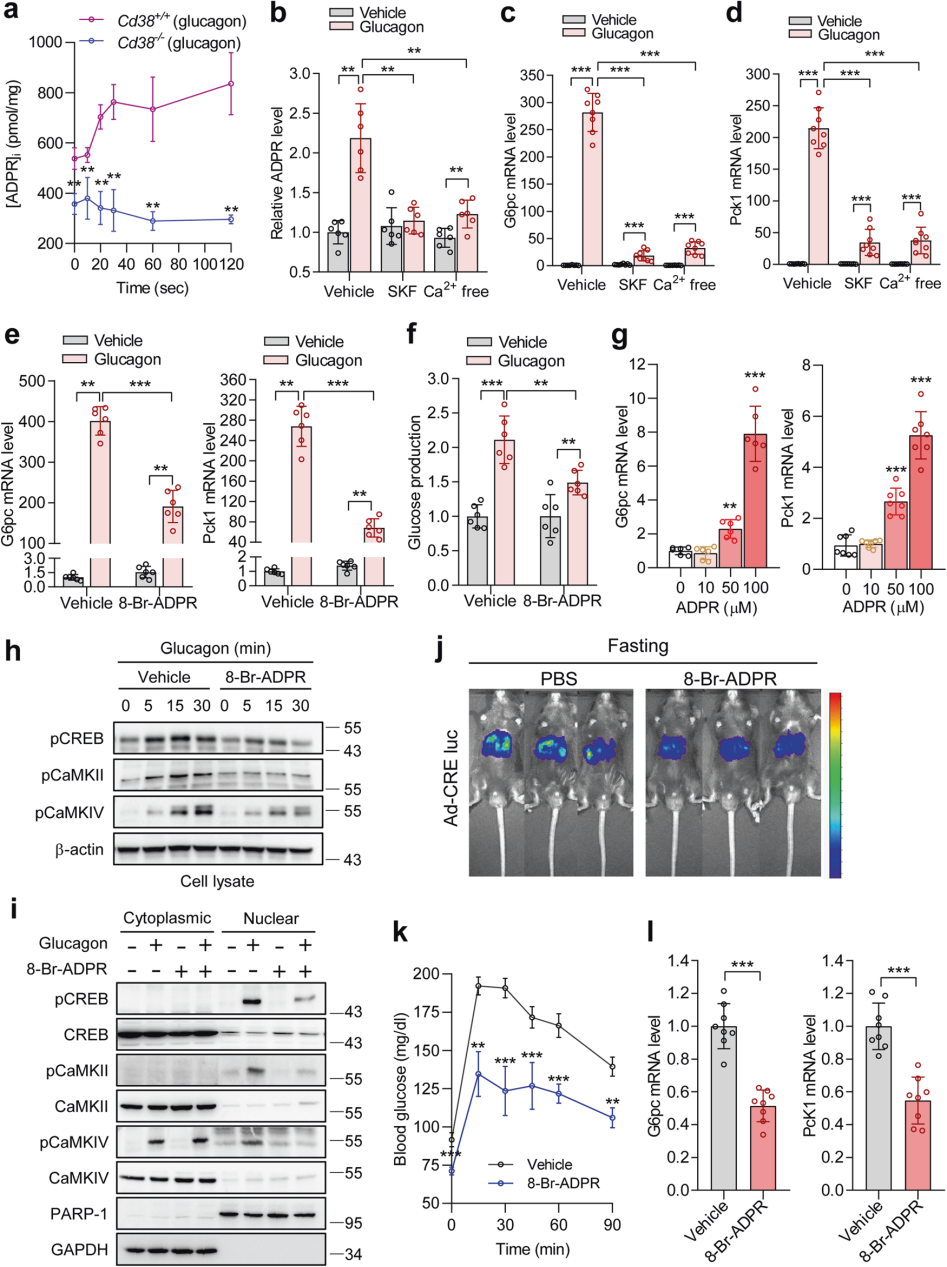
The CD38/ADPR-dependent nuclear Ca^2+^-CaMKII/CaMKIV-CREB pathway is required for glucagon-induced gluconeogenesis. **a** Glucagon-induced ADPR production in *Cd38*^*+/+*^ or *Cd38*^−*/*−^ primary hepatocytes. *n* = 6 independent experiments. **P < 0.01; *Cd38*^*+/+*^ vs. *Cd38*^*+/+*^. **b** ADPR levels in hepatocytes treated with glucagon (100 nM) for 30 sec after preincubation with vehicle or SKF 96365 (SKF, 50 μM), or under Ca^2+^-deprived extracellular conditions. *n* = 6 independent experiments. **P < 0.01. **c**, **d** Effects of SOCE on glucagon-induced mRNA levels of *G6pc* (**c**) and *Pck1* (**d**). *n* = 8 independent experiments. ***P < 0.001. **e**, **f** Effects of 8-Br-ADPR on glucagon-induced mRNA levels of *G6pc* (**e**, left) and *Pck1* (**e**, right) and glucose production (**f**). 8-Br-ADPR (100 μM) was preincubated for 30 min. *n* = 6 independent experiments. **P < 0.01, ***P < 0.001. **g** Effects of exogenous ADPR on *G6pc* and *Pck1* gene expression. *n* = 6 independent experiments. ***P < 0.001. **h** Immunoblotting for pCREB, pCaMKII, pCaMKIV, and β-actin in total cell lysates. **i** Immunoblotting cytoplasmic and nuclear extracts after treating hepatocytes with glucagon for 30 min. Cells were preincubated with 8-Br-ADPR (100 μM) for 30 min. The PARP-1 antibody was used as a marker of nuclear extracts. **j**, **k** Effects of 8-Br-ADPR on CRE luciferase activity in fasting mice (**j**, *n* = 3 mice per group) and the pyruvate tolerance test (**k***, n* = 5 mice per group). 8-Br-ADPR (32 mg/kg) was intravenously admi*n*istered to mice. **P < 0.01 and ***P < 0.001; vehicle vs. 8-Br-ADPR. **l** Effects of 8-Br-ADPR on *G6pc* and *Pck1* mRNA levels in fasting mice. *n* = 8 mice per group. ***P < 0.001. Data are represented as the mean ± SEM. Statistics were determined by unpaired *t* test (**a**, **g**, **k**, **l**) or one-way ANOVA followed by Tukey’s multiple comparison test (**b**–**f**). **h**, **i** Representative images of three independent experiments are shown.

In accordance with the data demonstrating ADPR as a potential messenger in nuclear Ca^2+^ signals, ADPR levels were increased in hepatocytes from *Cd38*^+/+^ mice at 20 sec in response to glucagon treatment (Fig. 5a), which correlates with the time course of glucagon-induced nuclear Ca^2+^ increase (Fig. 3a, b). As expected, hepatocytes from *Cd38*^−/−^ mice failed to produce ADPR in response to glucagon. Moreover, basal ADPR levels in hepatocytes from *Cd38*^−/−^ mice were also significantly lower than those in hepatocytes from *Cd38*^+/+^ mice (Fig. 5a). These findings indicate that CD38 is responsible for the glucagon-induced production of ADPR in hepatocytes. Pretreatment with 8-Br-cADPR completely blocked glucagon-induced ADPR production, while XeC only partially blocked ADPR production (Supplementary Fig. 4a, b). Thus, we tested whether 8-Br-cADPR inhibits glucagon-induced Cx43 phosphorylation, and as expected, it abolished glucagon-induced Cx43 phosphorylation (Supplementary Fig. 4c). Given that SOCE following IP_3_ and cADPR-mediated Ca^2+^ signals is critical for sustained Ca^2+^ signals and that 8-Br-ADPR blocks sustained nuclear Ca^2+^ signals (Fig. 3f), we considered the possibility that glucagon-induced ADPR production may require extracellular Ca^2+^ influx. This was confirmed by the observation that glucagon-induced ADPR production was blocked by SKF 96365 and extracellular Ca^2+^ deprived conditions (Fig. 5b). In contrast, glucagon-induced cADPR production was unaffected by SKF 96365 and extracellular Ca^2+^ deprivation (Supplementary Fig. 4d). In addition, pretreatment with SKF 96365 or extracellular Ca^2+^ free conditions inhibited the mRNA expression of *G6pc* and *Pck1* in response to glucagon (Fig. 5c, d). These results indicate that ADPR production by nCD38 requires SOCE, through which sustained nuclear Ca^2+^ signals, essential to glucagon-induced hepatic gluconeogenic gene expression, are enabled. Based on these results, we tested the effects of 8-Br-ADPR on gluconeogenesis. Pretreatment with 8-Br-ADPR significantly inhibited glucagon-induced gluconeogenic gene expression, as well as glucose production (Fig. 5e, f). We then tested whether exogenous ADPR increases gluconeogenic gene expression. Treating intact hepatocytes with ADPR increased gluconeogenic gene expression in a dose-dependent manner (Fig. 5g). Exogenous ADPR also increased glucose production (Supplementary Fig. 4e). Moreover, exogenous ADPR-induced gluconeogenic gene expression was unaffected by SKF 96365 and extracellular Ca^2+^ deprivation (Supplementary Fig. 4f). This result suggests that ADPR-mediated signaling is downstream of SOCE in gluconeogenic gene expression. Since CaMKII/CREB phosphorylation is required for hepatic gluconeogenesis in response to glucagon and nuclear Ca^2+^ signals induce the phosphorylation of CaMKIV/CREB in various cells^24,35,36^, we investigated whether ADPR-mediated nuclear Ca^2+^ signals were involved in CaMKII/CaMKIV/CREB phosphorylation in glucagon-treated hepatocytes. CaMKII/CaMKIV/CREB phosphorylation was induced as early as 5 min following glucagon treatment and was sustained until 30 min, which was inhibited by pretreatment with 8-Br-ADPR (Fig. 5h). Moreover, 8-Br-ADPR significantly inhibited glucagon-induced CaMKII/CaMKIV/CREB phosphorylation in the nucleus but not in the cytosol (Fig. 5i). Furthermore, the administration of 8-Br-ADPR reduced CRE luciferase activity as well as *G6pc* and *Pck1* mRNA expression levels in fasting mice, resulting in decreased blood glucose levels in both the fasting state and in response to a pyruvate challenge when compared with the control group (Fig. 5j–l and Supplementary Fig. 4g). However, the administration of 8-Br-ADPR to mice resulted in a decrease in body weight, white adipose tissue, plasma insulin, and hepatic glycogen content, with no change in plasma glucagon levels under fasting conditions (Supplementary Fig. 4h–k). Glucagon-induced cytosolic Ca^2+^ increase activates CaMKII to promote FoxO1 nuclear translocation through p38α, resulting in hepatic gluconeogenesis^36^. Because the inhibition of nuclear Ca^2+^ signaling by 8-Br-ADPR prevented the phosphorylation of CaMKII (Fig. 5h, i), we further examined whether glucagon-induced nuclear Ca^2+^ signaling by ADPR is involved in FoxO1 nuclear translocation. 8-Br-ADPR did not affect either glucagon-induced FoxO1 nuclear translocation or FoxO1 expression (Supplementary Fig. 5a, b). Because glucagon increased the expression of FoxO1, we further examined the effect of 8-Br-ADPR on glucagon-induced nuclear translocation of FoxO1 from the cytosol in the presence of cycloheximide, an inhibitor of protein synthesis. Although the inhibition of glucagon-induced nuclear Ca^2+^ signaling by 8-Br-ADPR prevented the phosphorylation of CaMKII (Fig. 5h, i), 8-Br-ADPR did not affect FoxO1 nuclear translocation (Supplementary Fig. 5c, d). Taken together, these data suggest that glucagon stimulates gluconeogenesis through nuclear Ca^2+^ signals increased by nuclear CD38/ADPR activity.

### PLCδ1/δ3 nuclear translocation by glucagon mediates nuclear Ca^2+^ increase and gluconeogenesis

**Fig. 6:**
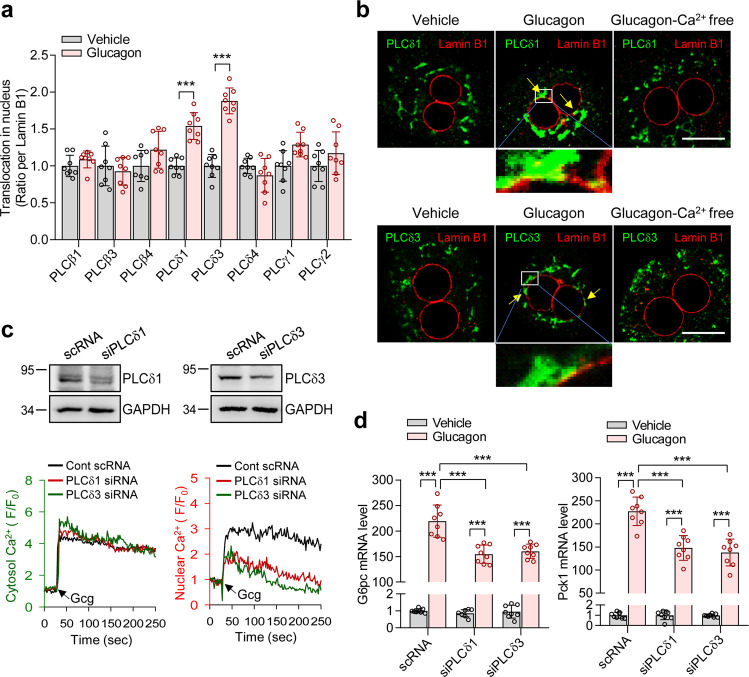
PLCδ1/δ3 nuclear translocation is required for glucagon-induced nuclear Ca^2+^ signals and gluconeogenesis. **a** Mean densitometric values in Fig. S5B. *n* = 8 independent experiments. ***P < 0.001. Data are represented as mean ± SEM. Statistics were determined by unpaired *t* test. **b** Immunostaining of PLCδ1 and PLCδ3. Glucagon (100 nM) was administered to hepatocytes for 30 sec. Scale bar, 10 μm. Representative images of three independent experiments are shown. **c** Immunoblot of PLCδ1, PLCδ3, and GAPDH in hepatocytes treated with control scRNA or PLCδ1 or PLCδ3 siRNA. Representative images of three independent experiments are shown. Representative cytosolic and nuclear Ca^2+^ responses to glucagon in control scRNA-, PLCδ1 siRNA-, or PLCδ3-treated hepatocytes. The time point where 100 nM glucagon (Gcg) is added is indicated by the arrow. *n* = 20 cells for each condition. **d** Glucagon-induced mRNA levels of *G6pc* and *Pck1* in control scRNA-, PLCδ1 siRNA-, or PLCδ3-siRNA treated hepatocytes. *n* = 8 indepe*n*dent experiments. ***P < 0.001. Data are represented as the mean ± SEM. Statistics were determined by one-way ANOVA followed by Tukey’s multiple comparison test.

Nuclei possess phosphoinositide signaling mechanisms that are similar to those that occur at the plasma membrane, which regulates the amounts of the membrane phospholipid phosphatidylinositol 4,5-bisphosphate [PtdIns(4,5)P_2_] and phosphoinositide-specific phospholipase C (PtdIns-PLC)^37–39^. Nuclear PtdIns-PLC acts as positive feedback for nuclear Ca^2+^ signaling^40^. Thus, we investigated whether the increase in the nuclear Ca^2+^ signal with glucagon is affected by nuclear PLC activation. First, we identified the expression of PLCβ1, β3, β4, δ1, δ3, δ4, γ1, and γ2 isoforms in mouse primary hepatocytes (Supplementary Fig. 6a). Although most PLC isoforms display cytoplasmic localization in the unstimulated state, some PLC isoforms translocate to the nucleus after agonist treatment^40^. Therefore, we examined which PLC isoforms translocate to the nucleus upon stimulation with glucagon. Glucagon treatment resulted in the nuclear translocation of PLCδ1 and PLCδ3 but not the PLCβ1, β3, β4, δ4, γ1, or γ2 isoforms (Fig. 6a and Supplementary Fig. 6b). PLCδ1 and PLCδ3 were distributed outside of the nucleus during the nonstimulated state, whereas after stimulation with glucagon, the PLCs accumulated in clumps in the nuclear membrane. This translocation took place only in the presence of Ca^2+^ (Fig. 6b). These findings are consistent with our data showing that glucagon-induced nuclear Ca^2+^ signals were abolished in the Ca^2+^-free state (Supplementary Fig. 3b). PLCδ1 and PLCδ3 involvement in glucagon-induced nuclear Ca^2+^ signals and gluconeogenic gene expression was confirmed by PLCδ1 and PLCδ3 KD experiments (Fig. 6c, d). In contrast, PLCδ1 and PLCδ3 KD did not affect glucagon-induced cytosolic Ca^2+^ signals. These results indicate that nuclear translocation of PLCδ1 and PLCδ3 by glucagon is important for nuclear Ca^2+^ signals and hepatic gluconeogenesis.

### ADPR-mediated nuclear Ca^2+^ signaling is required for gluconeogenesis in fasting mice and in a diabetic mouse model

**Fig. 7:**
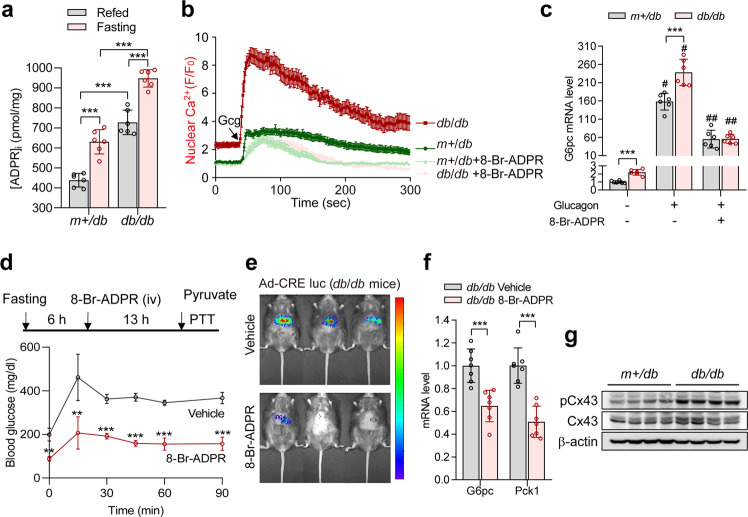
ADPR-mediated nuclear Ca^2+^ signaling modulates hepatic gluconeogenesis in fasting and diabetes. **a** Hepatic ADPR level of *m* + */db* and *db/db* mice fed and fasted for 19 h. *n* = 6 independent experiments. ***P < 0.001. **b** Effect of 8-Br-ADPR on glucagon-induced nuclear Ca^2+^ signaling in hepatocytes prepared from *m* + */db* and *db/db* mice. The time point where 100 nM glucagon (Gcg) is added is indicated by the arrow. *n* = 20 cells for each condition. **c** Glucagon-induced mRNA levels of *G6pc* in hepatocytes prepared from *m* + */db* and *db/db* mice. Cells were preincubated with 8-Br-ADPR (100 μM) for 30 min before treatment with glucagon (100 nM). *n* = 6 indepe*n*dent experiments. ***P < 0.001, ^#^*P* < 0.05; vehicle vs glucagon, ^##^*P* < 0.001; glucagon *vs* 8-Br-ADPR plus glucagon. **d**–**f** Effects of 8-Br-ADPR on the pyruvate tolerance test (**d**, *n* = 5 mice per group), CRE luciferase activity (**e***, n* = 3 mice per group), and gluconeogenic gene expression (**f**, *n* = 7 mice per group) in *db/db* mice. 8-Br-ADPR (32 mg/kg) was intravenously administered to mice. **P < 0.01, ***P < 0.001. **g** Immunoblotting for pCx43 (S368), Cx43, and β-actin in the livers of *m* + */db* and *db/db* mice after fasting for 19 h. *n* = 4 mice per group. Data are represented as the mean ± SEM. Statistics were determined by unpaired *t* test (**d**, **f**) or one-way ANOVA followed by Tukey’s multiple comparison test (**a**, **c**).

The increase in circulating glucagon promotes hepatic gluconeogenesis in the fasting state and in diabetes^5^. We also confirmed an increase in plasma glucagon levels in the fasting condition when compared to fed WT mice (Supplementary Fig. 7a). Therefore, we examined the effects of hepatic ADPR-mediated Ca^2+^ signaling on gluconeogenic activity during fasting and in diabetes. We measured ADPR levels in hepatocytes from *m* + */db* and diabetic animals during fasting as well as in the refed state. Hepatic ADPR levels were significantly higher in *db/db* mice than in *m* + */db* mice, and ADPR levels were higher during fasting than after refeeding in both *m* + */db* and *db/db* mice in correlation with plasma glucagon levels (Fig. 7a and Supplementary Fig. 7b, c, e). Hepatocytes prepared from *db/db* mice showed higher levels of glucagon-induced nuclear Ca^2+^ signals and gluconeogenic gene expression when compared to hepatocytes from *m* + */db* mice, which was reduced by 8-Br-ADPR (Fig. 7b, c), implicating the potential role of ADPR-mediated nuclear Ca^2+^ signals in the pathogenesis of diabetes in association with gluconeogenesis. Correspondingly, administering 8-Br-ADPR to *db/db* mice reduced blood glucose levels in response to pyruvate challenge, CRE luciferase activity, and gluconeogenic gene expression in response to fasting (Fig. 7d–f). However, the administration of 8-Br-ADPR to *db/db* mice decreased plasma insulin levels and hepatic glycogen content, with no change in plasma glucagon levels (Supplementary Fig. 7d–f). Intriguingly, *db/db* mice showed upregulated Cx43 phosphorylation compared to normal mice, reinforcing the idea that ADPR production is regulated through Cx43 phosphorylation (Fig. 7g). Furthermore, fasting stimulated Cx43 phosphorylation at Ser^368^ and gluconeogenic gene expression, whereas feeding reduced Cx43 phosphorylation and gluconeogenic gene expression (Supplementary Fig. 8). These results suggest that ADPR-mediated nuclear Ca^2+^ signaling is important for glucose production in the fasting state and that the downregulation of ADPR signaling reduces circulating glucose levels in diabetes.

## Discussion

In the current study, we examined the role of nCD38 in glucagon-induced gluconeogenesis in hepatocytes. Importantly, no previous study has investigated the dependence of gluconeogenic gene transcription on nCD38 in response to glucagon. Nuclear CD38 produces two Ca^2+^-mobilizing messengers in the nucleus: cADPR and ADPR, each with distinct roles in nuclear Ca^2+^ signaling. Adebanjo et al.^8^ previously proposed that CD38, with its catalytic site facing the nucleoplasm, may produce cADPR to activate RyR on the inner nuclear membrane to induce the initial Ca^2+^ increase. In contrast, we found that CD38 with its catalytic site facing the perinuclear space, forms ADPR to activate TRPM2 for the later phases of nuclear Ca^2+^ signaling. NAD^+^ is supplied *via* Cx43 from the nucleoplasm, and this mechanism is regulated by PKCδ-dependent phosphorylation in response to glucagon. ADPR then activates the TRPM2 channel, after which Ca^2+^ flows from perinuclear Ca^2+^ stores into the nucleoplasm, an essential process in regulating gluconeogenic gene expression. Consistently, all molecules involved in this signaling process, CD38, Cx43, TRPM2, and PKCδ, are localized to the inner membrane of the nucleus. Our results suggest that glucagon-induced sustained Ca^2+^ signals in both the nucleus and the cytosol are dependent on IP_3_ and cADPR (Supplementary Fig. 3a, d). Dantrolene, an inhibitor of the ryanodine receptor, inhibited glucagon-induced gene expression of *G6pc* and *Pck1* (Supplementary Fig. 9). This is consistent with previous findings that IP_3_- and cADPR-mediated cytosolic Ca^2+^ signals are involved in glucagon-induced hepatic gluconeogenesis^5,6^. In addition, ER Ca^2+^ depletion results in SOCE^41,42^. Thus, Ca^2+^ mobilization from ER Ca^2+^ stores, triggered by IP_3_- and cADPR, is critical for the SOCE mechanism, emphasizing the importance of SOCE not only in replenishing perinuclear/ER Ca^2+^ stores but also for sustaining the Ca^2+^ signals in the cytosol and the nucleus (Supplementary Fig. 3b, c). Importantly, our results suggest that sufficient Ca^2+^ repletion of perinuclear Ca^2+^ stores through SOCE is a prerequisite for ADPR production by nCD38.

Nuclear Ca^2+^ signaling is regulated by nuclear PtdIns-PLC^40^. We also found that PLCδ1/δ3 are specifically involved in nuclear, but not cytosolic, Ca^2+^ signaling in response to glucagon stimulation. Previous findings have demonstrated that PLCδ1 translocates into the nucleus in a Ca^2+^-dependent manner^43^. Subsequently, this study demonstrates that IP_3_- and cADPR-mediated Ca^2+^ signals in the cytosol, which appear to be essential for the later cytosol and nuclear Ca^2+^ signals, trigger the nuclear translocation of PLCδ1/δ3. Inhibition of these cytosolic and nuclear Ca^2+^ signals with 8-Br-cADPR blocked ADPR production by inhibiting Cx43 phosphorylation, affirming that ADPR production is tightly regulated by upstream Ca^2+^ signaling.

It has been reported that PKCδ KO mice (either global or liver-specific) displayed increased hepatic insulin signaling and reduced expression of gluconeogenic enzymes^44^. Consistent with this report, we showed that the PKCδ inhibitor or PKCδ knockdown reduced glucagon-induced expression of *G6pc* and *Pck1* (Fig. 2i, j). Our data suggest that PKCδ may be a key regulator of gluconeogenesis. As mentioned earlier, we found that upstream signaling of the nuclear translocation of PLCδ1/δ3 activates nuclear PKCδ, which phosphorylates Cx43 to gate NAD from the nucleoplasm into the perinuclear space (Fig. 2k–m). Given that constitutively active catalytic sites of type II CD38 are compartmentalized with limited substrate availability^45^, nCD38 can produce ADPR by using the supplied NAD in the perinuclear space. ADPR activates the TRPM2 Ca^2+^-release channel to increase nuclear Ca^2+^, ultimately resulting in the expression of *G6pc* and *Pck1* (Fig. 2).

Glucagon regulates glucose homeostasis via a cAMP/PKA-dependent signaling pathway that results in the phosphorylation of CREB, which in turn activates the transcription of gluconeogenic enzymes^46^. Moreover, PKA mediates the phosphorylation of IP_3_R to increase cytosolic Ca^2+^, leading to an increase in calcineurin activity and the subsequent dephosphorylation of the CREB coactivator CRTC2^5^. Based on our results, ADPR-mediated nuclear Ca^2+^ signaling is essential for CaMKII/CaMKIV/CREB phosphorylation, and nuclear Ca^2+^ signals are a determinant factor, converging multiple upstream signals, including cytosolic Ca^2+^ signaling, in gluconeogenic gene transcription in response to glucagon. This conclusion is supported by findings that the upregulation of ADPR-mediated Ca^2+^ signaling results in increased gluconeogenesis in diabetes.

**Fig. 8:**
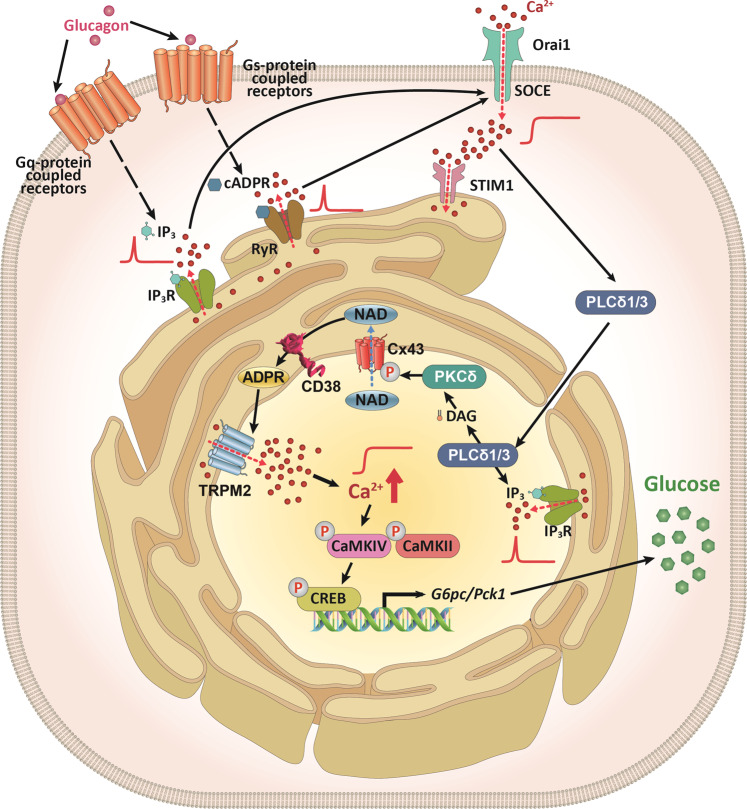
Schematic model: glucagon promotes gluconeogenesis via the nCD38-ADPR-Ca^2+^ signaling pathway. Glucagon regulates glucose homeostasis by binding to two G protein-coupled receptors (Gq and Gs) to produce IP_3_ and cADPR, respectively. IP_3−_ and cADPR-mediated Ca^2+^ mobilization from ER Ca^2+^ stores induces SOCE, resulting in Ca^2+^ influx. This activates the nuclear translocation of PLCδ1/3. Nuclear PLCδ1/3 produces IP_3_ and DAG, activating PKCδ, which phosphorylates Cx43. Phospho-Cx43 gates NAD from the nucleoplasm into the perinuclear space. Constitutively active CD38, with its catalytic site facing the perinuclear space, produces ADPR, which then activates TRPM2 to trigger nucleoplasmic Ca^2+^ release. Nuclear Ca^2+^ increases the expression of *G6pc* and *Pck1*, which promote gluconeogenesis through the CaMKII/CaMKIV/CREB pathway. Note that CD38, Cx43, TRPM2, and PKCδ are all located on the inner nuclear membrane.

In summary, this study has identified the essential role of nCD38 in glucagon-treated primary hepatocytes (Fig. 8). The compartmentalization of CD38 in the nucleus increases nuclear Ca^2+^ influx through TRPM2 activated by ADPR, leading to activation of the CaMKII/CaMKIV/CREB pathway for gluconeogenesis. We suggest that nCD38 plays a critical role in glucose homeostasis under physiological conditions such as fasting.

## Data and materials availability

All data needed to evaluate the conclusions in the paper are present in the main text or the supplementary materials.

## Supplementary information

Supplementary information
