# Supplementary material for: CD38/ADP-ribose/TRPM2-mediated nuclear Ca2+ signaling is essential for hepatic gluconeogenesis in fasting and diabetes
Source: Exp Mol Med. 2023 Jul 3;55(7):1492–505. doi: 10.1038/s12276-023-01034-9 (PMC10393965; doi:10.1038/s12276-023-01034-9)
Supplement: Supplementary file 1 — Supplementary information [file 12276_2023_1034_MOESM1_ESM.pdf]

Supplementay information

CD38/ADP-ribose/TRPM2-mediated nuclear Ca^{2+} signaling is essential for hepatic gluconeogenesis in fasting and diabetes

So-Young Rah[1], Yeonsoo Joe[2], Jeongmin Park[2], Stefan W. Ryter[3], Chansu Park[1], Hun Taeg Chung[2, *], and Uh-Hyun Kim[1, 4, *]

[1]Department of Biochemistry and National Creative Research Laboratory for Ca^{2+} signaling Network, Jeonbuk National University, Medical School, Keum-am dong, Jeonju, 54907, Republic of Korea. [2]School of Biological Sciences, University of Ulsan, Ulsan, 44610, Republic of Korea.[3]Proterris Inc., Boston, MA, USA, 02118. [4]Department of Biochemistry, School of Medicine, Wonkwang University, Iksan, 54538, Republic of Korea.

*Corresponding author. Email: uhkim@jbnu.ac.kr (U.-H.K), chung@ulsan.ac.kr (H.T.C)

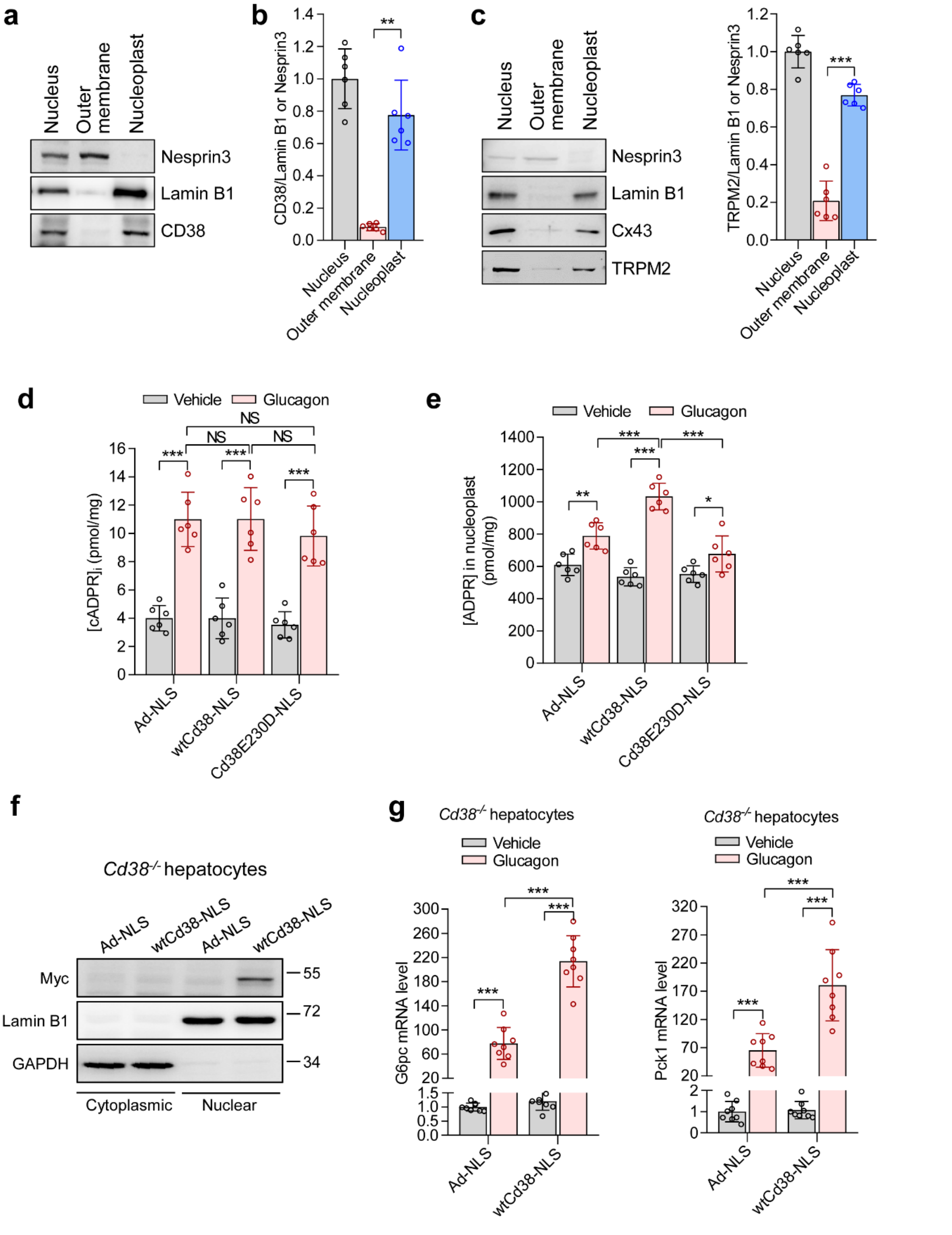

Supplementary Fig. 1. Cx43, CD38, and TRPM2 are located in the inner membrane of hepatocyte nuclei. a-c After isolating nuclei, the outer membrane of the nuclei was separated

with sodium citrate treatment. Immunoblot analysis was performed regarding CD38, Cx43, TRPM2, Nesprin3 (a marker for the outer membrane of the nucleus) and Lamin B1 (a marker for the inner membrane of the nucleus) in fractions containing intact nucleus, the outer membrane of the nucleus, and nucleoplasts, respectively. The bar graph represents the localization of CD38 (b) and TRPM2 (c). Data are represented as mean ± SEM. *n* = 6 independent experiments. $^{**}P < 0.01$, $^{***}P < 0.001$. **d** cADPR levels in hepatocytes treated with glucagon (100 nM) for 30 sec after infection with adenoviral vectors expressing Ad-NLS, wtCd38-NLS, or Cd38E230D-NLS at a MOI of 50 for 24 h. Data are represented as mean ± SEM. *n* = 6 independent experiments. $^{***}P < 0.001$ and not significant (NS). **e** ADPR levels in nucleoplasts. Data are represented as mean ± SEM. *n* = 6 independent experiments. $^{*}P < 0.05$, $^{**}P < 0.01$, $^{***}P < 0.001$. **f** Immunoblots of cytoplasmic and nuclear extracts about Myc, LaminB1, and GAPDH after overexpression of nCD38 in $Cd38^{-/-}$ hepatocytes using Ad-wtCd38-NLS. Representative images of three independent experiments are shown. **g** Glucagon-induced mRNA levels of *G6pc* and *Pck1* after overexpression of nCD38 in $Cd38^{-/-}$ hepatocytes using Ad-wtCd38-NLS. Data are represented as mean ± SEM. *n* = 6 independent experiments. $^{***}P < 0.001$. Statistical significance was determined by unpaired t test (b and c) or one-way ANOVA followed by Tukey’s multiple comparison test (d, e, and g).

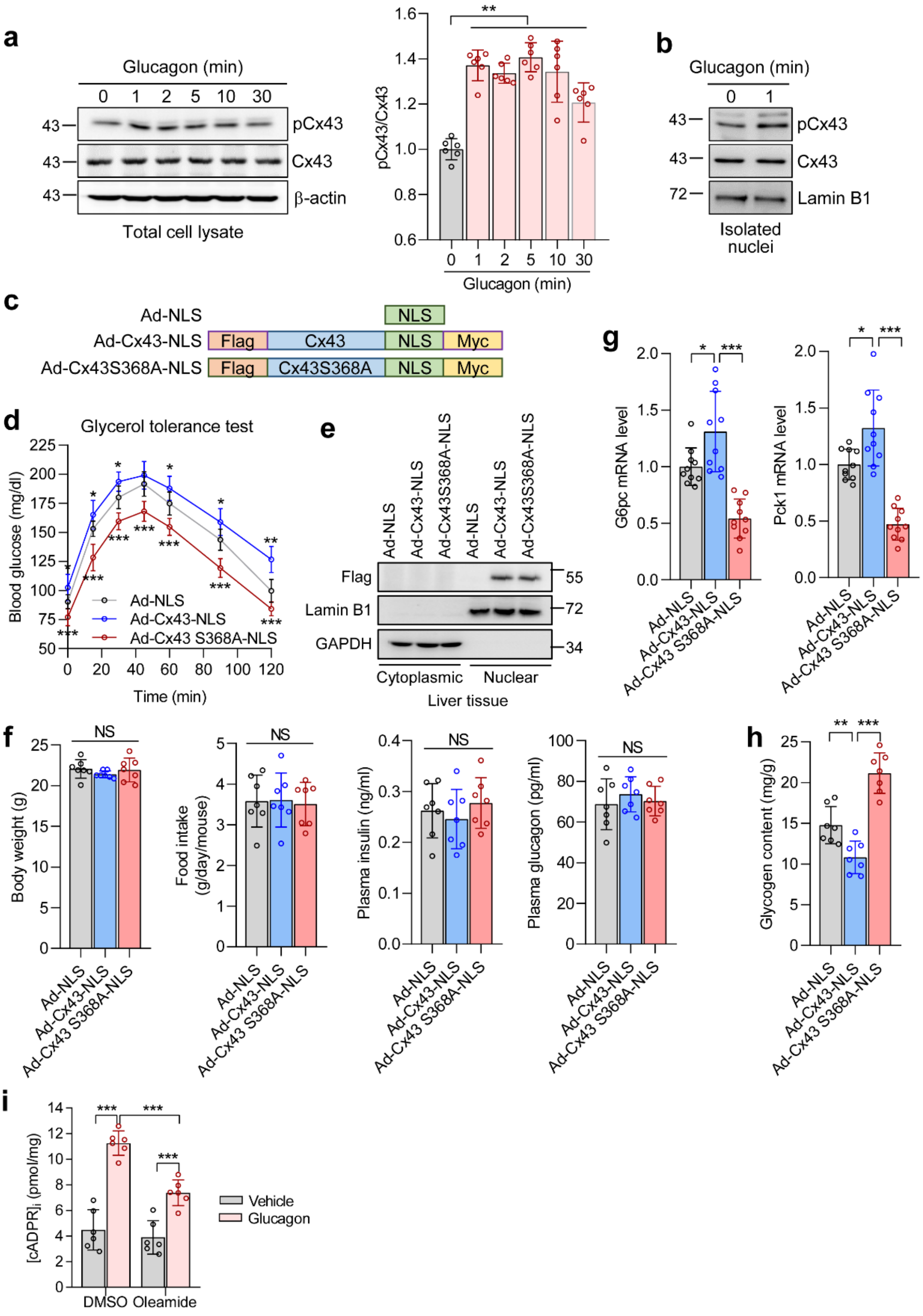

Supplementary Fig. 2. Glucagon-induced phosphorylation of Cx43 is involved in gluconeogenesis. a Immunoblotting about pCx43 (S368), Cx43, and β-actin. Mean

densitometric values of glucagon-induced Ser^{368} phosphorylation of Cx43. Data are represented as mean ± SEM. $n = 6$ independent experiments. **$P < 0.01$. Statistical significance was determined by unpaired t test. **b** Intact nuclei were treated with glucagon (100 nM) for 1 min. Immunoblotting about pCx43 (S368), Cx43, and Lamin B1. Representative images of three independent experiments are shown. **c** Adenoviral vector constructs for the nuclear targeting of Control (Ad-NLS), Cx43 (Ad-Cx43-NLS), and Cx43S368A (Ad-Cx43S368A-NLS). **d** Glycerol tolerance tests in mice after treatment with Ad-NLS or Ad-Cx43-NLS or Ad-Cx43S368A-NLS. $n = 7$ mice per group. *$P < 0.05$ and **$P < 0.01$; Ad-NLS *vs.* Ad-Cx43-NLS, ***$P < 0.001$; Ad-Cx43-NLS *vs.* Ad-Cx43S368A-NLS. **e** Immunoblotting with cytoplasmic and nuclear extracts from liver tissues after treatment with Ad-NLS or Ad-Cx43-NLS or Ad-Cx43S368A-NLS. Representative images of three independent experiments are shown. **f** Body weight, food intake, and levels of plasma insulin and glucagon in fasting mice treated with Ad-NLS or Ad-Cx43-NLS or Ad-Cx43S368A-NLS. $n = 7$ mice per group and not significant (NS). **g** Hepatic *G6pc* and *Pck1* mRNA levels in fasting mice treated with Ad-NLS or Ad-Cx43-NLS or Ad-Cx43S368A-NLS. $n = 10$ mice per group, *$P < 0.05$, ***$P < 0.001$. **h** Hepatic glycogen levels. $n = 7$ mice per group. **$P < 0.01$, ***$P < 0.001$. **i** Effect of oleamide on glucagon-induced cADPR production. Data are represented as mean ± SEM. $n = 6$ independent experiments. ***$P < 0.001$. Statistical significance was determined by one-way ANOVA followed by Tukey's multiple comparison test (d, f, g, h and i).

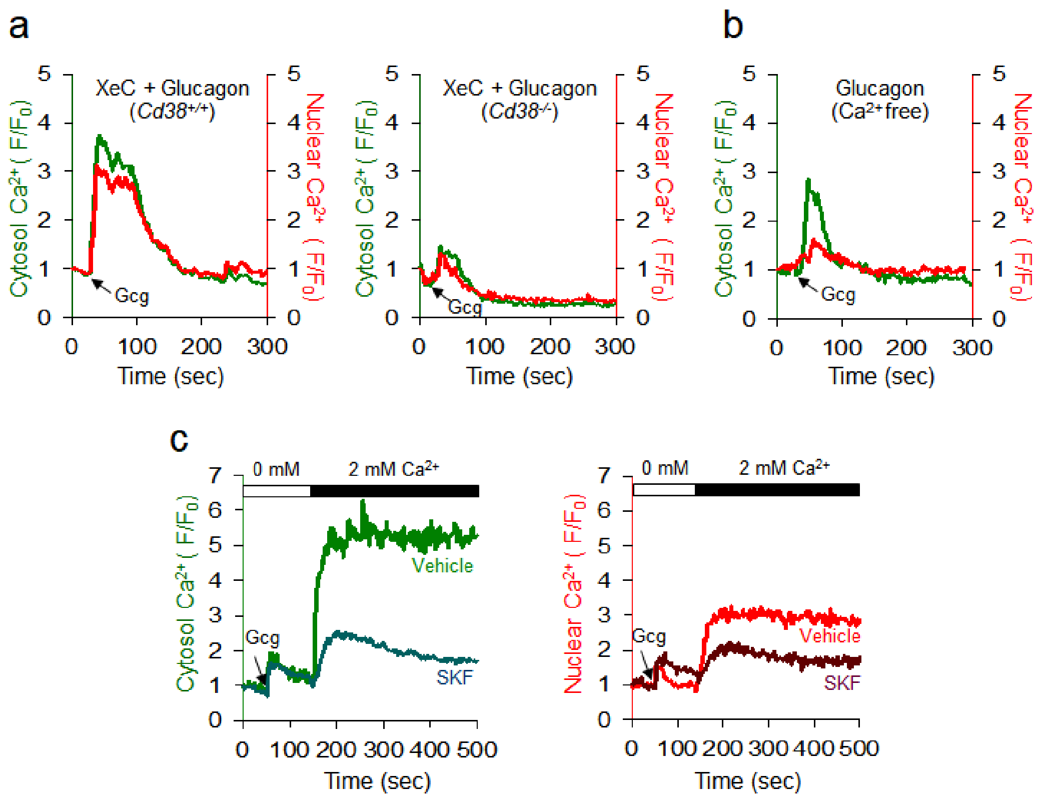

Supplementary Fig. 3. CD38/ADPR-dependent glucagon-induced nuclear Ca^{2+} increase in mouse primary hepatocytes. a Cytosolic and nuclear Ca^{2+} responses to glucagon after preincubation with XeC (2 μM) for 30 min in *Cd38* $^{+/+}$ and *Cd38* $^{-/-}$ primary hepatocytes. **b** Cytosolic and nuclear Ca^{2+} responses to glucagon under Ca^{2+} deprived extracellular conditions. **c** Glucagon-induced cytosolic and nuclear Ca^{2+} signals are regulated by SOCE. In the absence of external Ca^{2+}, the addition of glucagon depleted the Ca^{2+} stores, causing a Ca^{2+} release to the cytosol and the nucleus. External 2 mM Ca^{2+} induced sustained Ca^{2+} signals in the cytosol and the nucleus, which were blocked by SKF96365 (SKF, 50 μM) treatment. The time point where 100 nM glucagon (Gcg) is added is indicated by the *arrow*. *n* = 20 cells for each condition.

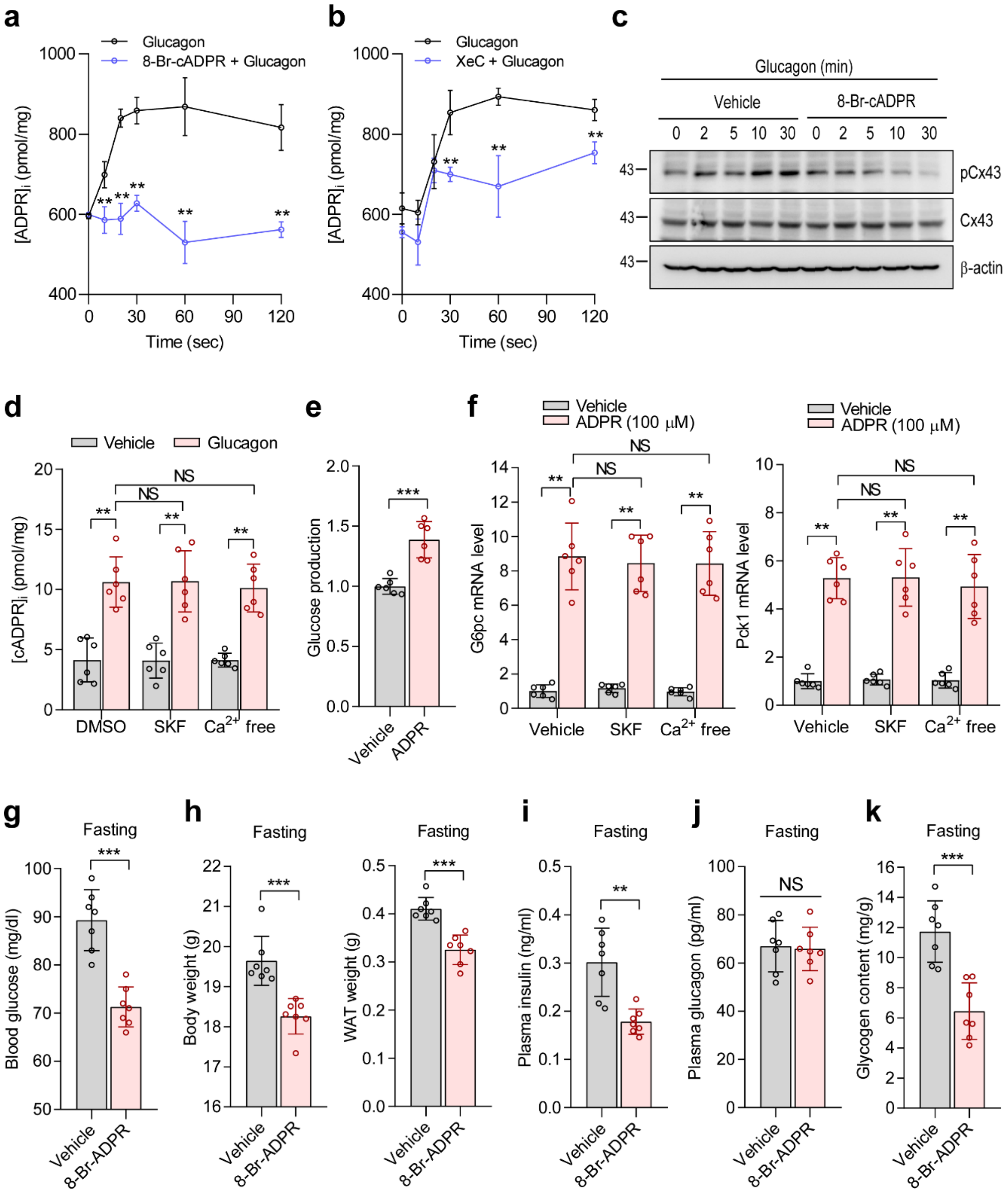

Supplementary Fig. 4. ADPR-induced gluconeogenesis is regulated by Ca^{2+}. a, b Effects of 8-Br-cADPR or XeC on glucagon-induced ADPR production. Primary hepatocytes were preincubated with 8-Br-cADPR (100 μM) or XeC (2 μM) for 30 min and then treated with glucagon for the indicated times. ADPR levels were measured using LC-MS/MS. n = 4

independent experiments. **$P < 0.01$, glucagon *vs* 8-Br-ADPR plus glucagon or XeC plus glucagon. **c** 8-Br-cADPR inhibits glucagon-induced phosphorylation of Cx43. Hepatocytes were treated with glucagon for the indicated times, lysed, and analyzed *via* immunoblot analysis using antibodies against pCx43 (S368), Cx43, and β-actin. Cells were preincubated with 8-Br-cADPR for 30 min before treatment with glucagon. Representative images of three independent experiments are shown. **d** cADPR levels in hepatocytes treated with glucagon (100 nM) for 30 sec after preincubation with a vehicle, or SKF 96365 (SKF, 50 μM), or under Ca^{2+} deprived extracellular conditions. $n = 6$ independent experiments. **$P < 0.01$ and not significant (NS). **e** Effects of exogenous ADPR on glucose production. Glucose output was measured after the treatment with 100 μM ADPR for 5 h. $n = 6$ independent experiments. ***$P < 0.001$. **f** Effects of SKF 96365 or extracellular Ca^{2+} deprivation on exogenous ADPR-induced gluconeogenic gene expression. $n = 6$ independent experiments. **$P < 0.01$ and not significant (NS). **g-k** Blood glucose (g), body weight and WAT (white adipose tissue) weight (h), plasma insulin (i) and plasma glucagon levels (j), and hepatic glycogen content (k) in fasting mice treated with 8-Br-ADPR (32 mg/kg). $n = 7$ mice per group. **$P < 0.01$, ***$P < 0.001$, and not significant (NS). Data are represented as mean ± SEM. Statistical significance was determined by unpaired t test (a, b, e, g, h, i, j and k) or one-way ANOVA followed by Tukey's multiple comparison test (d and f).

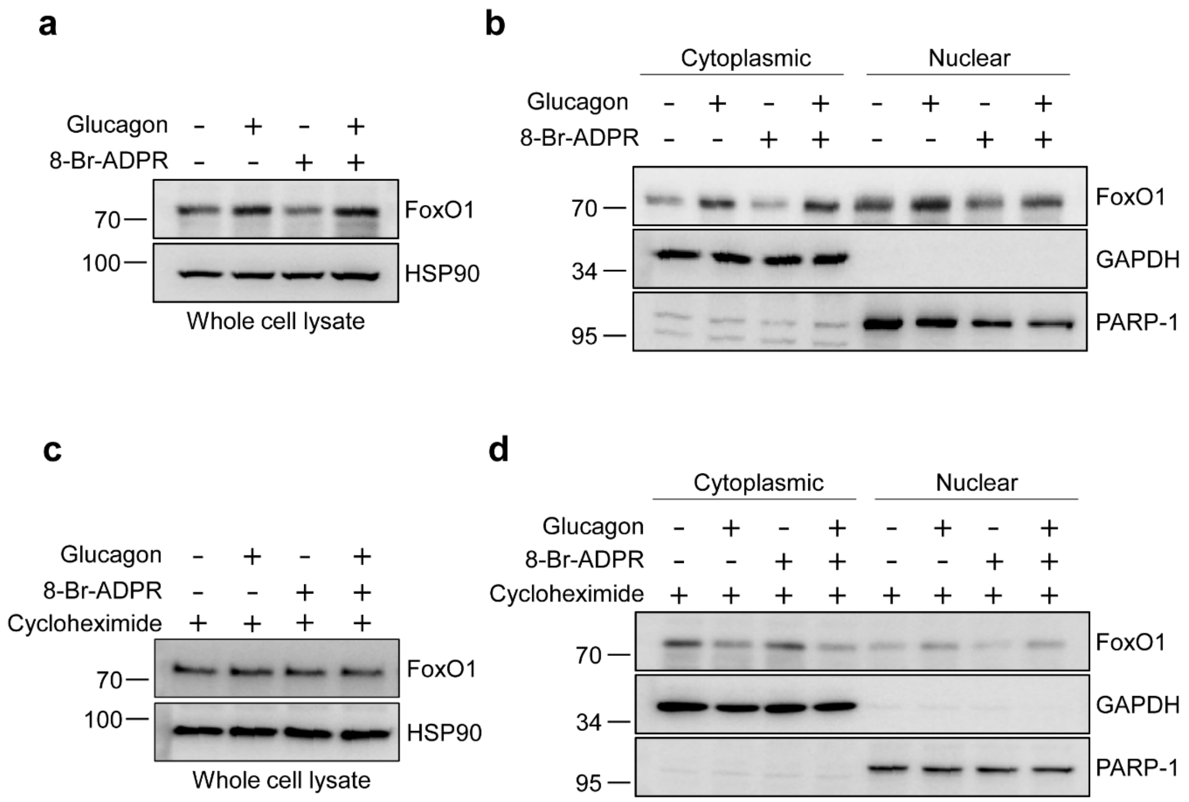

Supplementary Fig. 5. Effects of 8-Br-ADPR on glucagon-induced FoxO1 nuclear translocation. a Glucagon-induced FoxO1 protein expression. 8-Br-ADPR (100 μM) was preincubated for 30 min before the treatment with glucagon (100 nM) for 4 h. **b** Immunoblotting about cytoplasmic and nuclear extracts after treating hepatocytes with glucagon (100 nM) for 4 h. 8-Br-ADPR (100 μM) was preincubated for 30 min. FoxO1 localization was analyzed by immunoblot using antibodies against each marker: GAPDH (a marker of cytoplasmic extracts) and PARP-1 (a marker of nuclear extracts). **c, d** Effects of cycloheximide on glucagon-induced FoxO1 protein expression (c) and FoxO1 nuclear treansloation (d). Cycloheximide (10 μg/ml) was preincubated for 30 min to prevent protein synthesis before the treatment with glucagon (100 nM) for 4 h. Representative images of three independent experiments are shown.

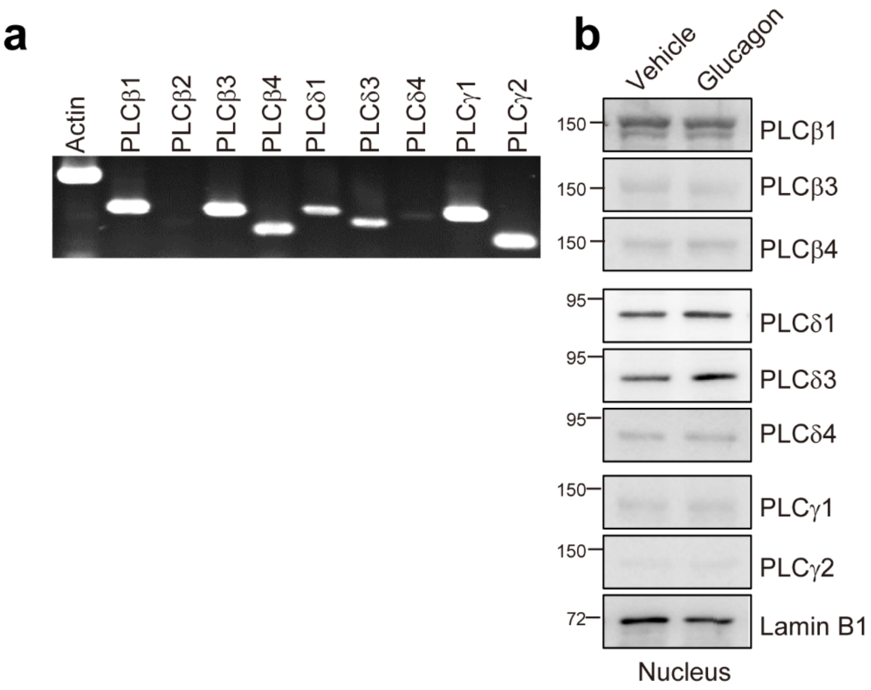

Supplementary Fig. 6. Glucagon-induced PLCδ1/δ3 translocation to nucleus. a RT-PCR about PLC isoforms in mouse primary hepatocytes. **b** Glucagon induces translocation of PLCδ1/δ3 to nuclear. After the treatment with glucagon for 30 sec in hepatocytes, nuclus was isolated. Immunoblotting about PLCδ isoforms and Lamin B1. n = 8 independent experiments. Representative images are shown.

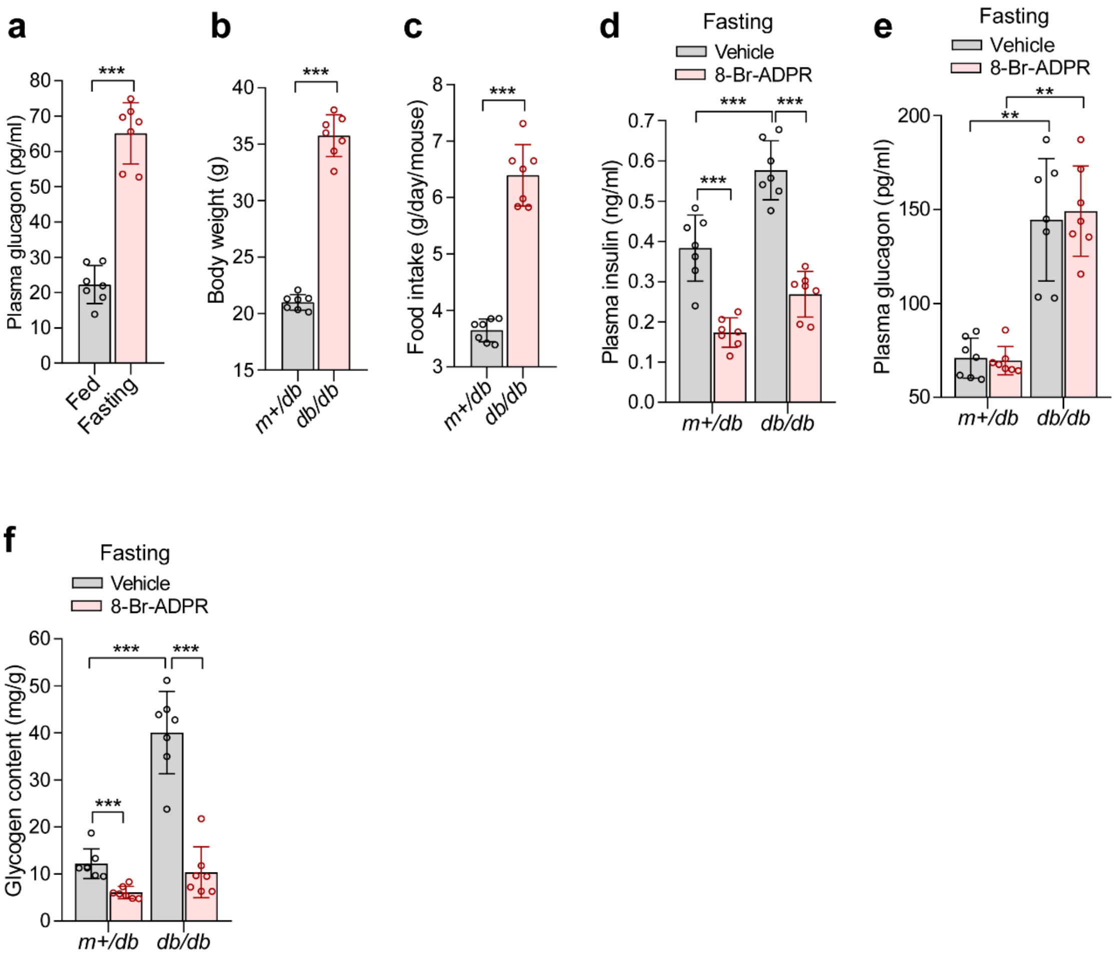

Supplementary Fig. 7. Effects of 8-Br-ADPR in diabetic mice. a Plasma glucagon levels in fed and fasted WT mice. **b, c** Body weight and food intake in *m+/db* and *db/db* mice. **d-f** Levels of plasma insulin (d), plasma glucagon (e), and hepatic glycogen content (f) in *m+/db* and *db/db* mice treated with 8-Br-ADPR (32 mg/kg). $n = 7$ mice per group. $^{**}P < 0.01$, $^{***} P < 0.001$. Data are represented as mean ± SEM. Statistical significance was determined by unpaired t test (a, b, and c) or one-way ANOVA followed by Tukey's multiple comparison test (d, e, and f).

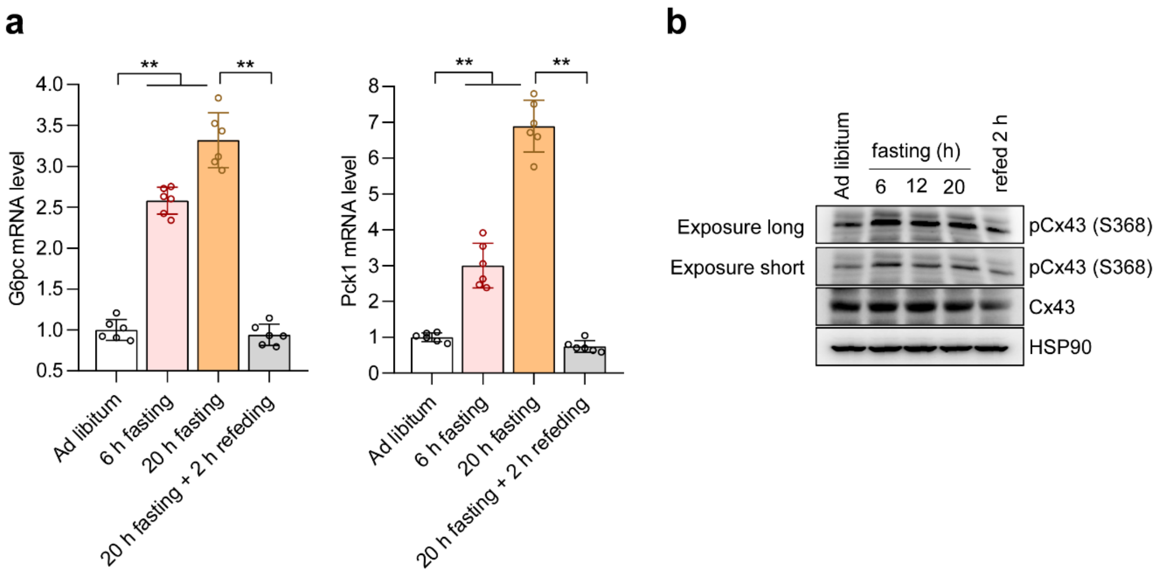

Supplementary Fig. 8. Phosphorylation of Cx43 was increased in fasting condition. a, b Liver tissues fasted for the indicated times or refed for 2 h after fasting for 20 h were lysed and used for qRT-PCR of *G6pc* and *Pck1* (a), and for immunoblot analysis using antibodies against pCx43 (S368), Cx43, and HSP90 (b). n = 6 mice per group. **P < 0.01. Data are represented as mean ± SEM. Statistical significance was determined by one-way ANOVA followed by Tukey's multiple comparison test. For b, representative images of three independent experiments are shown.

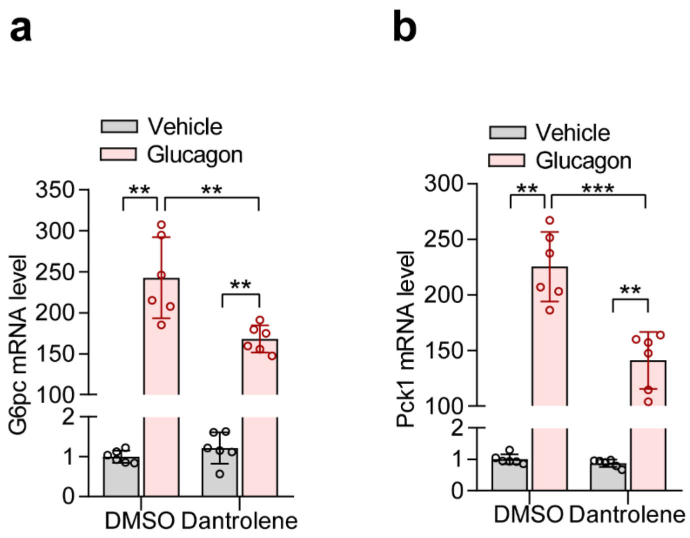

Supplementary Fig. 9. Effects of Dantrolene on glucagon-induced *G6pc* and *Pck1* gene expression. qRT-PCR of *G6pc* (a) and *Pck1* (b) after incubation with glucagon (100 nM) for 4 h. Dantrolene (10 μM), an inhibitor of ryanodine receptor, was preincubated for 1 h. $n = 6$ independent experiments. $^{**}P < 0.01$. Data are represented as mean ± SEM. Statistics were determined by one-way ANOVA followed by Tukey's multiple comparison test.
